# Exploring chronic and transient tumor hypoxia for predicting the efficacy of hypoxia-activated pro-drugs

**DOI:** 10.1038/s41540-023-00327-z

**Published:** 2024-01-05

**Authors:** Shreya Mathur, Shannon Chen, Katarzyna A. Rejniak

**Affiliations:** 1 H. Lee Moffitt Cancer Center and Research Institute, IMO High School Internship Program, Tampa, FL USA; 2 H. Lee Moffitt Cancer Center and Research Institute, Integrated Mathematical Oncology Department, Tampa, FL USA; 3 University of South Florida, Morsani College of Medicine, Department of Oncologic Sciences, Tampa, FL USA; 4 Present Address: University of Florida, Undergraduate Studies, Gainesville, FL USA

**Keywords:** Cancer, Applied mathematics

## Abstract

Hypoxia, a low level of oxygen in the tissue, arises due to an imbalance between the vascular oxygen supply and oxygen demand by the surrounding cells. Typically, hypoxia is viewed as a negative marker of patients’ survival, because of its implication in the development of aggressive tumors and tumor resistance. Several drugs that specifically target the hypoxic cells have been developed, providing an opportunity for exploiting hypoxia to improve cancer treatment. Here, we consider combinations of hypoxia-activated pro-drugs (HAPs) and two compounds that transiently increase intratumoral hypoxia: a vasodilator and a metabolic sensitizer. To effectively design treatment protocols with multiple compounds we used mathematical micro-pharmacology modeling and determined treatment schedules that take advantage of heterogeneous and dynamically changing oxygenation in tumor tissue. Our model was based on data from murine pancreatic cancers treated with *evofosfamide* (as a HAP) and either *hydralazine* (as a vasodilator), or *pyruvate* (as a metabolic sensitizer). Subsequently, this model was used to identify optimal schedules for different treatment combinations. Our simulations showed that schedules of HAPs with the vasodilator had a bimodal distribution, while HAPs with the sensitizer showed an elongated plateau. All schedules were more successful than HAP monotherapy. The three-compound combination had three local optima, depending on the HAPs clearance from the tissue interstitium, each two-fold more effective than baseline HAP treatment. Our study indicates that the three-compound therapy administered in the defined order will improve cancer response and that designing complex schedules could benefit from the use of mathematical modeling.

## Introduction

Solid tumors often have an inefficient blood supply for a prolonged time, leading to the emergence of regions within tumors that are poorly oxygenated, a condition known as chronic hypoxia. The oxygen levels in these areas can be 5 to 10 times lower than in normal tissues^1,2^. This is a cumulative effect of limited vascular perfusion, perturbed interstitial diffusion, and increased oxygen demand from rapidly proliferating and expanding tumor cells. As a result, steep oxygen gradients and a diffusion-limited hypoxia have been observed in these tumors^2,3^. This physiological tissue state has been associated with the development of aggressive tumors with metastatic capabilities, and resistance to chemo-, immuno-, and radiation therapies^4–6^. To overcome the physical barriers created by intratumor hypoxia, chemotherapeutic compounds have been developed, known as the hypoxia-activated pro-drugs (HAPs). HAPs are chemically active only in the areas of low oxygen levels where they release high-dose chemotherapy and remain non-lethal in the well-oxygenated regions^7–10^. Several HAPs have been included in clinical trials, either as monotherapy or in combination with other drugs or radiation therapy^11–13^, although they have not been as successful as expected^14,15^.

This mathematical study’s research question of interest is how to enlarge the area of HAP activation by inducing transient hypoxia. This can temporarily increase the regions of low oxygen contents, but should regress to its initial state after a couple of hours. In general, the spatial distribution of oxygen within the tissue depends on three factors: oxygen vascular supply, the cellular uptake of oxygen, and oxygen interstitial transport. In the present study, we considered two ways for the transient increase of tumor hypoxic regions: decreasing the oxygen influx and increasing oxygen absorption. In the first method we use vasodilators, which reduce the blood flow into the tumor vessels and thus diminish the influx of oxygen to the tumor tissue. The use of vasodilators leads to the dilation and increased perfusion of healthy blood vessels, which may result in reduced blood flow in vasculature parallel to healthy tissues, such as tumor vasculature; this process is known as the vascular steal phenomenon^16^. As a consequence, the oxygen perfusion in tumor tissues is diminished, and intratumoral hypoxia is elevated^17^. In the second method we use metabolic sensitizers^13,18^ to elevate the uptake of oxygen by the tumor cells and thus temporarily increase the extent of hypoxia within the tissue. We refer here to experimental data that used *hydralazine* as a vasodilator, *pyruvate* as a metabolic sensitizer, and *evofosfamide* (also known as *TH-302*) as a hypoxia-activated pro-drug, with *bromo-isophosphoramide mustard* (Br-IPM) as its active metabolite, in mice experiments with Mia PaCa-2 pancreatic tumors.

Since the behavior of HAPs is complex (as their activation depends on the metabolic landscape of the tumor microenvironment, which in turn can be modulated by the effects of HAP action), it is difficult to test drug combinations with HAP experimentally in a controlled manner. Moreover, when the combinations of HAPs with two other compounds are considered, determining the right drug sequence and injection timing is beyond what is feasible with empirical testing. Therefore, we utilized our mathematical micro-pharmacology modeling^19^ to examine various multi-treatment schedules and determine optimal dosing regimens that would maximize the number of dead cells. The Results section includes description of simulation design, simulations of HAP monotherapy, the combination of HAP with the vasodilator, the combination of HAP with the sensitizer, as well as optimization of the three-compound combination therapy. Conclusions are presented in the Discussion section. The mathematical model is defined in the Methods section.

## Results

In this section, we present (i) the design of our micro-pharmacology model and the overall strategy to analyze the effects of combination therapies for enhancing the efficacy of HAPs; (ii) the simulated changes in intratumoral oxygen distribution after a bolus injection of HAP; (iii) the transient effects of an application of the vasodilator; (iv) schedule optimization of the vasodilator in combination with HAP; (v) the transient effects of an administration of the metabolic sensitizer; (vi) computational scheduling optimization of the sensitizer in combination with HAP; and (vii) the optimization of the three-compound combination therapy.

### General model design

Our mathematical micro-pharmacokinetics/pharmacodynamics (microPKPD) model^19,20^ uses a digitized tumor histology image from a pancreatic tumor cell line Mia PaCa-2 as a computational domain for all model simulations. This digitized tissue includes the explicitly defined vasculature along the left edge of the domain, a non-uniform architecture of the tumor cells, and the irregular extracellular space (the interstitium), all of which affects the transport of the interstitial fluid and all diffusive compounds. Oxygen is supplied by tumor vasculature and is absorbed by the cells, resulting in the formation of an oxygen gradient and the emergence of hypoxic regions. The HAP *evofosfamide* (also known as *TH-302*) is supplied from the vasculature in an inactive form and then activated upon reaching the hypoxic areas within the tissue. Only the activated drug is absorbed by the cells to exert the lethal effect. To temporarily increase the regions of drug activation, we considered intravenous injection of an additional pharmacological compound, either the vasodilator *hydralazine* or the metabolic sensitizer *pyruvate*, or both. The sensitizer resulted in higher oxygen absorption (in a dose-dependent manner) by cells that were exposed to it. The vasodilator decreased blood flow in the tumor vasculature, and thus diminished the influx of oxygen, the inactive drug, and the sensitizer, if they were present in circulation at the same time as the vasodilator.

**Fig. 1:**
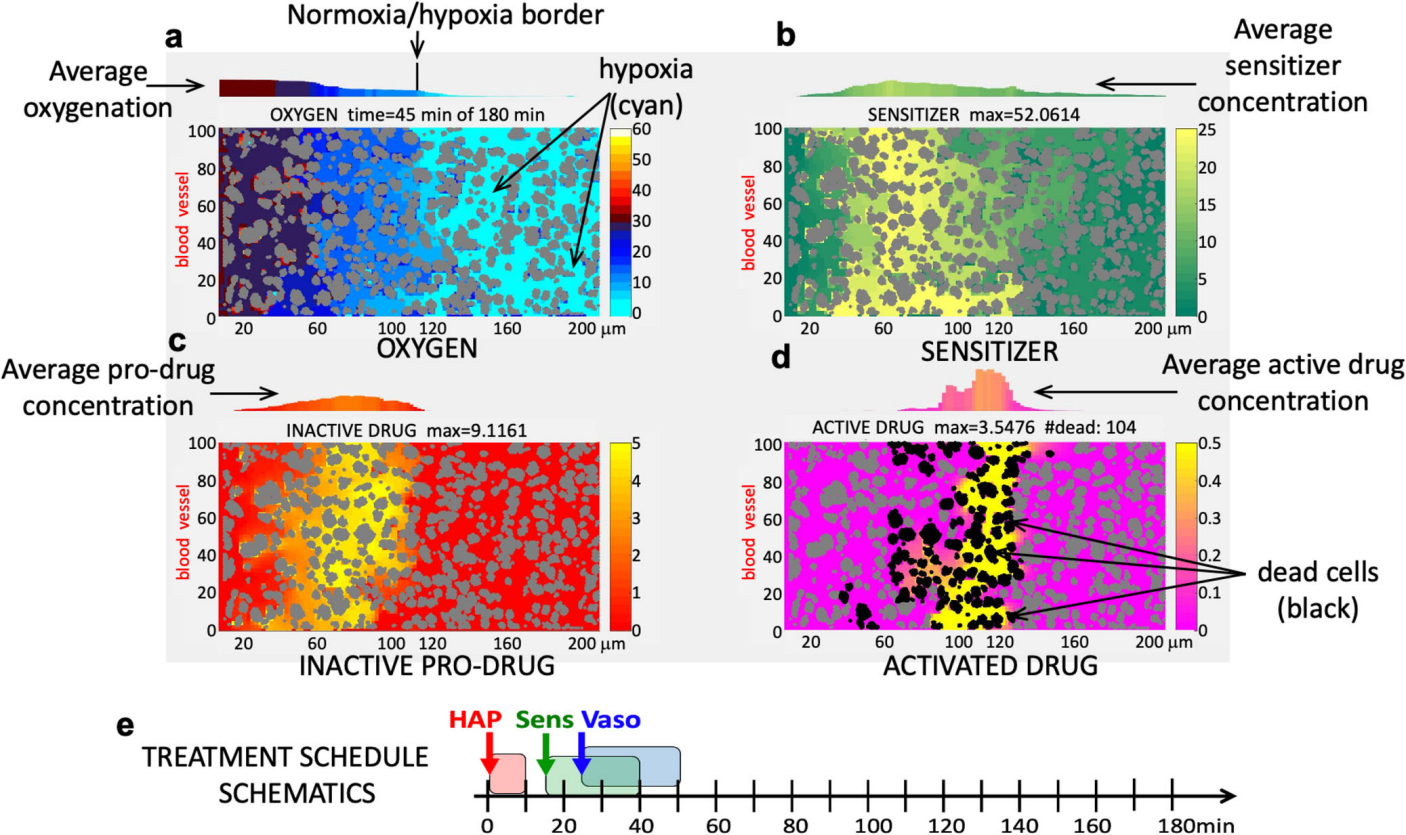
Design of model outcome panels. **a** Spatial distribution of oxygen with hypoxic regions indicated in cyan and the normoxia/hypoxia border shown by a vertical line. **b** Spatial distribution of a metabolic sensitizer. **c** Spatial distribution of an inactive pro-drug. **d** Spatial distribution of an activated drug with dead cells shown in black. **b**-**d** High concentrations of each compound are shown in yellow with a corresponding scale bar (different for each compound) shown to the right. **e** Schematics of a typical treatment schedule with bolus injections of the hypoxia-activated drug (HAP), a sensitizer (Sens), and a vasodilator (Vaso).

All model outcomes are presented using four panels, as shown in Fig. 1, each depicting the same tissue morphology with cells in gray, a single blood vessel located along the left boundary of the domain, and the distribution of one of the following compounds penetrating the tumor tissue: (a) oxygen either under regular influx or modulated by the vasodilator (Vaso), (b) metabolic sensitizer (Sens), (c) hypoxia-activated pro-drug in its inactive form (HAP), and (d) the effector drug (i.e., the activated form of HAP). To better visualize gradients of each diffusible factor the corresponding scales of compound concentration are different (shown on the right of each panel). The average compound concentrations along the tissue length, shown above each tissue panel, were calculated as an average value from a vertical tissue strip below; this presentation helps in visual quantification of the spread of each compound. In particular, the average oxygenation graph includes a vertical line that indicates a border between the well-oxygenated and the hypoxic areas of the tissue (the normoxia/hypoxia border). In addition, the active drug panel shows the dead tumor cells (in black), that is, those cells that have absorbed lethal amounts of the active drug.

The overall strategy to analyze all mono- and combination therapies may be summarized as follows: (i) simulate HAP kinetics within the tissue based on published *evofosfamide* data; (ii) simulate the kinetics of a vasodilator (Vaso) alone and in combination with HAP, based on published *hydralazine* and *evofosfamide* data; (iii) design optimal treatment protocols for the HAP+Vaso combination; (iv) simulate kinetics of a sensitizer (Sens) alone and in combination with HAP based on published *pyruvate* and *evofosfamide* data; (v) design optimal treatment protocols for the HAP+Sens combination; (vi) design optimal treatment protocols for the combination of all three treatments (HAP + Vaso + Sens). The measure of treatment efficacy is the ratio of dead to viable tumor cells after 3 hours of the simulated treatment.

### Oxygen landscape under HAP exposure

**Fig. 2:**
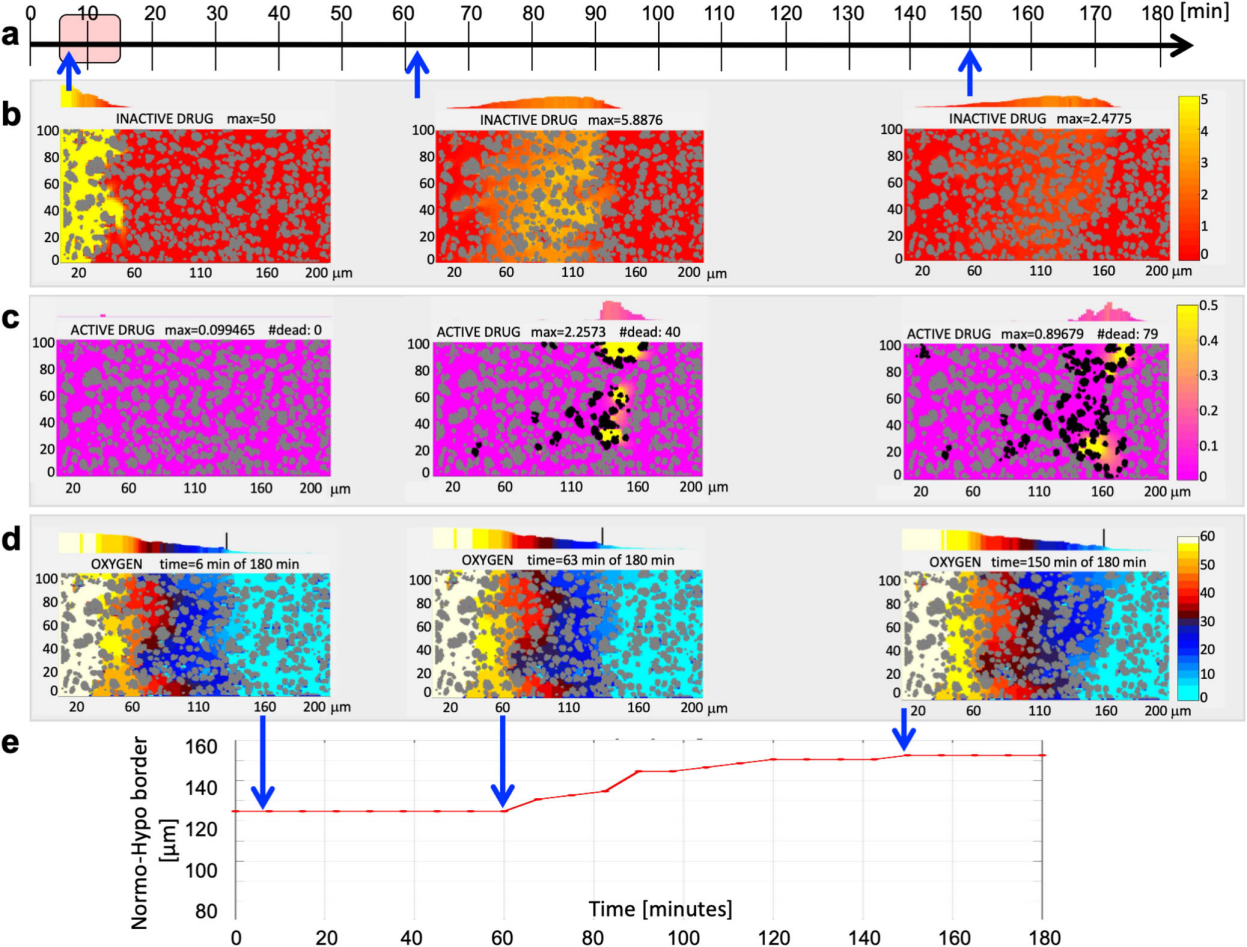
Shift in tissue oxygen profile after exposure to HAP. **a** Treatment schedule with a single bolus injection of HAP between 5 and 15 minutes. **b** Spatial distribution of the inactive HAP at 6, 63, and 150 minutes. **c** Corresponding spatial distribution of the active drug. **d** Corresponding spatial distribution of oxygen. The scale bars (different for each compound) are shown to the right. **e** The change of normoxia/hypoxia border distance from the vessel over the time of simulation.

First, we examined the effects of HAP injection on the tumor and its microenvironment. In a typical mice experiment, a bolus injection of 50 *μg/ml* of HAP is administered intravenously^21^. This drug was observed in the blood stream for about 10 minutes until it had cleared from the mouse body^21^. These data were used to calibrate HAP vascular concentration of ${\eta }_{i}^{0}=50$ *ag/μm*^3^ (after rescaling) as well as time of HAP plasma clearance, ${t}_{\eta i}=10\min$. During the time of HAP circulation, the drug extravasated the vascular system and penetrated the tissue via both diffusion and advection. The HAP diffusion coefficient was estimated to be ${D}_{\eta i}={D}_{\gamma }$/50, based on *evofosfamide* molecular weight. The interstitial fluid influx velocity ${{\boldsymbol{u}}}_{{in}}=1\mu m/s$ was based on published experimental data^22,23^. Once the HAP reached the hypoxic areas (the oxygen level of drug activation was $10{mmHg}$,^24^), it was converted to its active form. We assumed 90% conversion rate to preserve the relationship between *TH-302* and *Br-IPM* concentrations from Fig. 4 of^21^ that shows an order of magnitude difference between them. We followed this indirect evidence, since no direct data on the conversion rate from *TH-302* to its active metabolite *Br-IPM* is known. The active drug was again transported via diffusion (with a diffusion coefficient ${D}_{\eta a}=2{D}_{\eta i}$ due to its smaller size) and advection (the same as for the non-active drug). The active drug was also absorbed by the tumor cells with an uptake rate $\alpha =0.5/\min$, and it was a subject to decay with a decay rate ${\omega }_{a}=\log (2)/10$ per minute. Once the cell accumulated the active drug above the lethal concentration ${\eta }_{a}^{{thr}}=1{ag}/\mu {m}^{3}$ ^21^, it was assumed dead; as a result, this cell stopped absorbing both oxygen and the active drug. The corresponding simulation outcome is shown in Fig. 2 and a more detailed set of snapshots in Supplementary Fig. 1. Model parameter sensitivity analysis for the HAP monotherapy is shown in Supplementary Fig. 2.

After a single bolus injection of HAP that was present in the blood system over a period of $10\min$ (Fig. 2a), the inactive drug extravasated from the vessel and penetrated the tumor tissue (Fig. 2b). Shortly after injection, no active drug was present in the tissue, since HAP had not yet reached the hypoxic areas. At the later stages, the active drug was absorbed by tumor cells in the hypoxic areas and killed cells that had accumulated the lethal amount of the active drug (the black cells in Fig. 2c). Since the dead cells stopped absorbing the active drug, it could penetrate more deeply into the tumor tissue. Because the dead cells also stopped absorbing oxygen, the patterns of oxygen distribution changed over time, leading to reoxygenation of some areas that were previously hypoxic (Fig. 2d). This scenario was also reflected in the calculated normoxia/hypoxia border (Fig. 2e), referring to the distance at which the average oxygenation in the vertical tissue strip reached the hypoxic value for the first time. The initial normoxia/hypoxia border of about $124\mu m$ remained constant until the first dead cells emerged at about 60 minutes; after that, this distance steadily increased, eventually reaching a plateau of $152\mu m$ after about 150 minutes of exposure to the HAP. At the end of 3 hours period, $23.8 \%$ (87 of 365) cells had died. This simulation constitutes our baseline result.

### Transient effect of hydralazine vasodilator

Because the tissue near the vessel was well-oxygenated, the intravascularly injected HAP could not be activated in this area, and no dead cells were observed in the vessel vicinity. Methods for temporarily increasing the extent of hypoxia and thus the area of HAP activation, are considered here. One of such methods is to use vasodilators to reduce the blood flow into the tumor vessels and to diminish the influx of oxygen to the tumor tissue. Vasodilators lead to dilation of healthy blood vessels and their increased perfusion, which may result in a reduced blood flow in vasculature parallel to healthy tissues, such as tumor vasculature; this process is known as the vascular steal phenomenon^16^. As a consequence, the oxygen perfusion in tumor tissues is diminished and intratumoral hypoxia is elevated--the presence of steal phenomenon has been confirmed by Bailey et al.^17^ in a murine model of pancreatic Mia PaCa-2 cancers in response to *hydralazine*. Their study also showed decreased tumor oxygenation after administration of hydralazine and increased efficacy of the combined hydralazine and *TH-302* treatment when compared to *TH-302* alone.

**Fig. 3:**
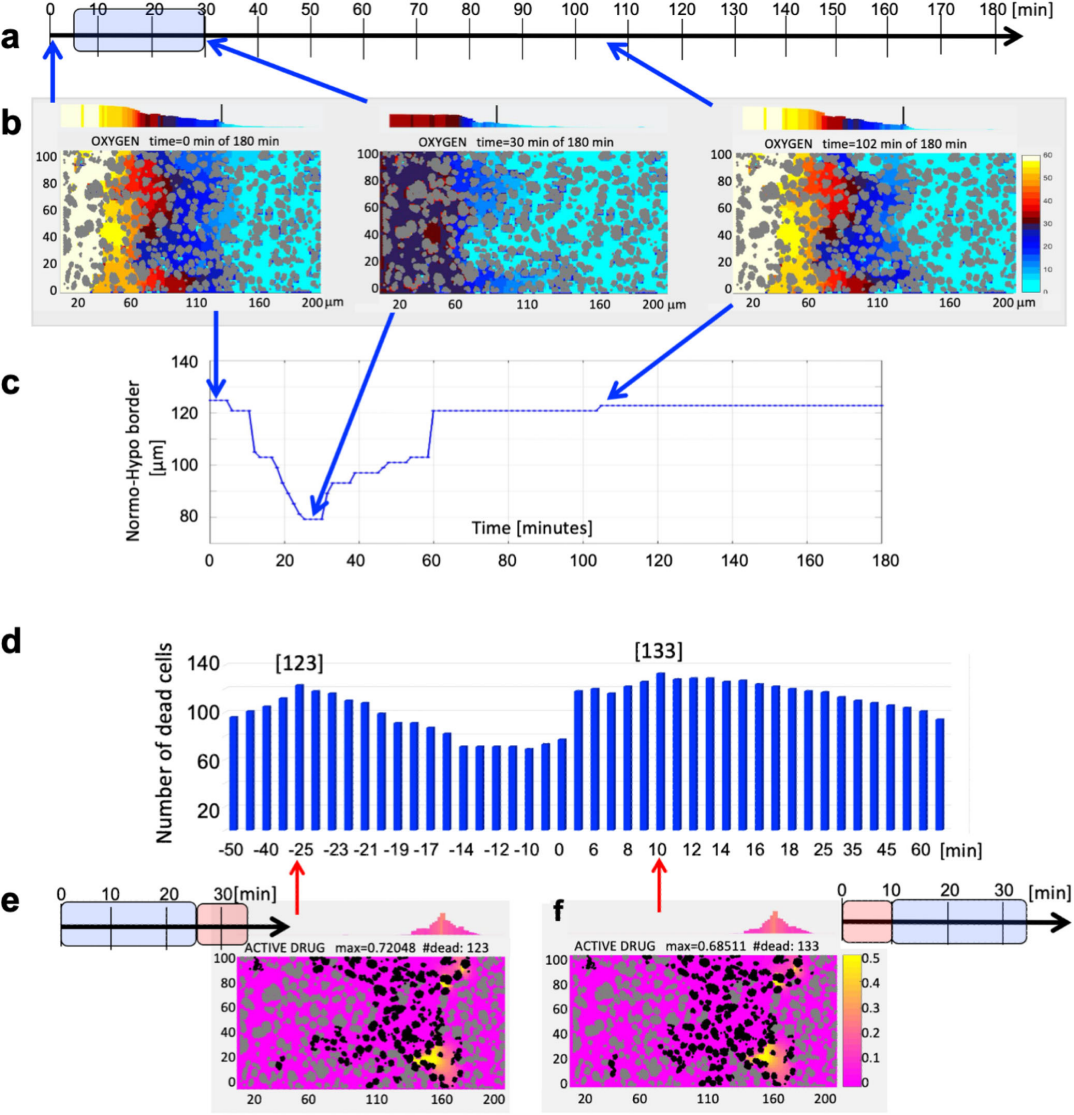
Vasodilator effect on tissue oxygenation and HAP activity. **a** A schedule for a bolus injection of vasodilator (Vaso). **b** Transient reduction in the oxygen influx into the tissue and the recovered the initial O_2_ level within an hour. **c** The change of the hypoxia/normoxia border for this single bolus injection of Vaso. **d** Effect (number of dead cells) of a bolus injection of Vaso before (- time) or after (+time) the HAP injection with two local maxima indicated by red arrows. **e** Final configuration of 123 dead cells for Vaso applied 25 minutes before HAP. **f** Final configuration of 133 dead cells for Vaso applied 10 minutes after HAP. **b, e, f** The corresponding scale bars (different for each compound) shown to the right.

Our mathematical model was parameterized with experimental data from^17^. Measurements with OxyLite needle electrodes at a single site inside the tumor tissue showed decrease in tumor oxygenation levels after administration of the vasodilator (Fig. 2d in^17^). However, these results were quite variable between 5 animals tested (oxygen reduction between 30% and 100%). Thus we modified the model boundary condition along the vessel and used an average value of 50% drop in the oxygen influx (Eq. (15), $\lambda =0.5$). For simplicity, we assumed that all reduction rates for all blood-borne compounds are identical. Moreover, the bolus injection of the vasodilator also reduced the blood flow into the tumor vessel by half (Fig. 2c in^17^). The minimal level of blood flow in the experiment was observed at 30 minutes, and blood flow recovery occurred between 1.5 to 2 hours^17^. We achieved the same effect by simulating the reduced oxygen influx for 25 minutes (Fig. 3a). This step led to the depletion of oxygen within the tissue, with the minimum reached at 30 minutes and the return to initial levels within 2 hours (Fig. 3b). We quantified these results by tracing the normoxia-hypoxia border (Fig. 3c), which is indicated by vertical lines in Fig. 3b. We thus were able to reproduce the induction of transient hypoxia within the tumor tissue by modulating the oxygen vascular influx.

### Optimizing HAP activity areas by modulating oxygen influx

**Fig. 4:**
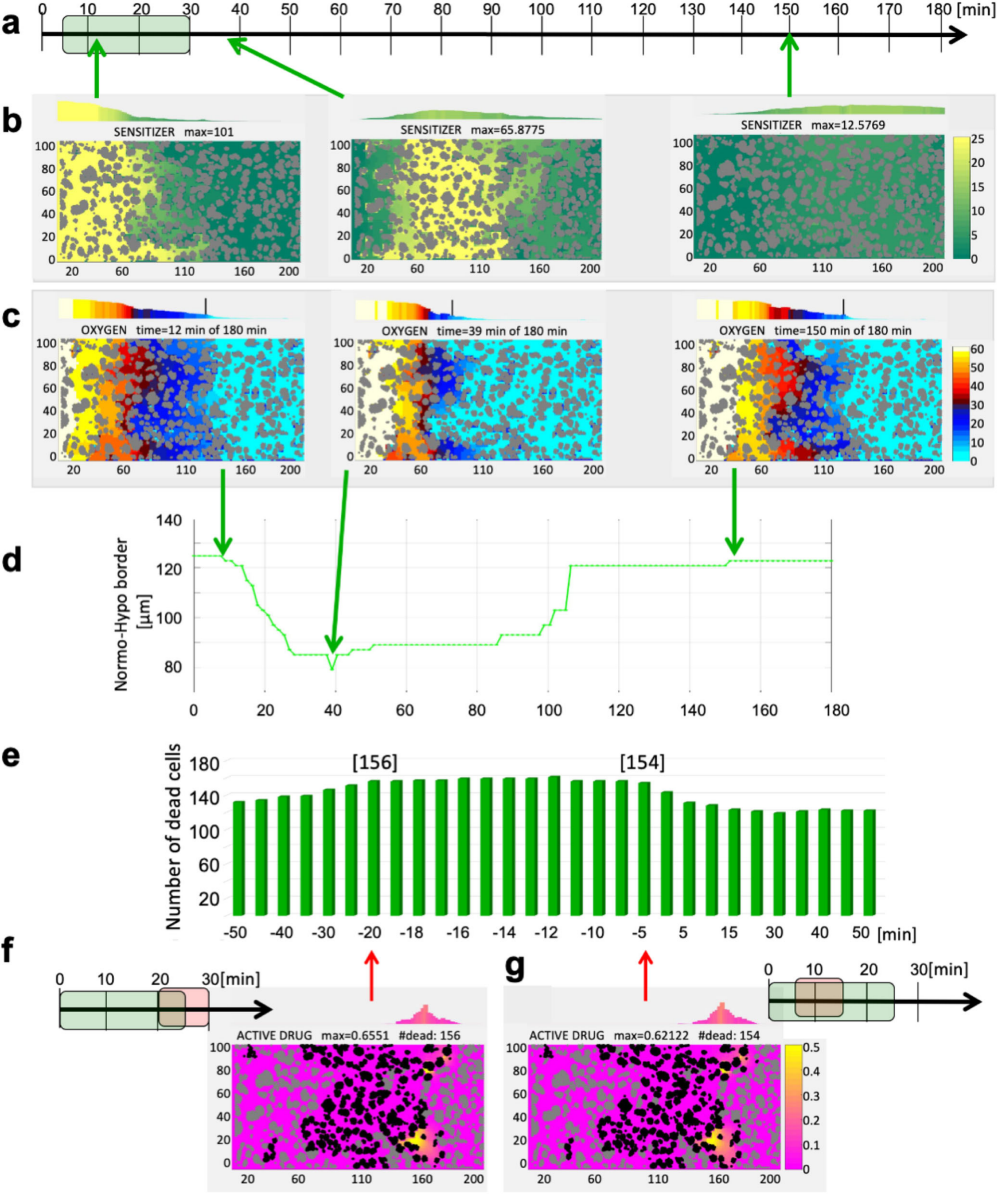
Sensitizer effect on tissue oxygenation and HAP activity. **a** A schedule for a bolus injection of the sensitizer (Sens). **b** Cellular exposure to the sensitizer gradient that transiently increases oxygen uptake by the cells and recovery of the initial O_2_ level within two hours (**c**). **d** The change of the hypoxia/normoxia border for this single bolus injection of Sens. **e** Effect (number of dead cells) of a bolus injection of Sens before (- time) or after (+time) the HAP injection with a significant plateau indicated by red arrows. Final configurations of dead cells for Sens applied 20 minutes (**f**) and 5 minutes (**g)** before HAP. **b, c, f, g** The corresponding scale bars (different for each compound) shown to the right.

Experimental studies reported by Bailey et al.^17^ showed that a combined therapy of *hydralazine* and *TH-302* reduced the growth of Mia PACa-2 tumors in mice (Fig. 4e in^17^), and prolonged mice life (Fig. 4f in^17^) when compared to TH-302 alone. In these experiments *hydralazine* was administered 30 minutes before *TH-302*^17^. In our study, we explored a wide range of schedules in order to maximize the number of dead cells. We simulated several schedules, with the vasodilator applied either before or after the HAP. In both cases the injection timing was crucial. For example, if the HAP was injected during the time the vasodilator was still present in the bloodstream, the vascular steal phenomenon would reduce the influx of both oxygen and HAP. Figure 3d shows the results of a series of computational simulations indicating the number of dead cells (out of 365) after 3 hours of the simulated time. The x-axis shows the time of vasodilator injection in respect to HAP injection time; the negative numbers represent cases where the vasodilator was injected before HAP (in minutes), while positive numbers represent the time of vasodilator injection after HAP. These results show the bimodal distribution of the number of dead cells with two local maxima: in the first schedule, the vasodilator was injected 25 minutes before HAP; in the second schedule, the vasodilator was injected 10 minutes after HAP. Figure 3e and Fig. 3f show the spatial distributions of dead cells in each case.

In the case, where the vasodilator was applied before the HAP, hypoxia has emerged first due to reduced oxygen influx and the subsequently injected inactive HAP encountered lower oxygen levels after entering the tissue. The HAP area of activation was much wider in this case than for HAP administered as a monotherapy. The most successful schedule was when HAP was injected just after the vasodilator was cleared from the bloodstream, since this approach allowed for HAP injection on full capacity, and oxygen concentration in the tissue had not yet returned to normal levels. At the end of this simulation, $33.7 \%$ (123 of 365) of cells had died (Fig. 3e). In the second case, the vasodilator was injected 10 minutes after the HAP. This approach allowed the inactive HAP to penetrate the tissue before the oxygen influx was reduced by the vasodilator. As a result, hypoxia emerged in the areas where the inactive HAP was already distributed, leading to its wider activation. At the end of this simulation, $36.44 \%$ (133 of 365) of cells had died (Fig. 3f). More detailed set of snapshots is shown in Supplementary Fig. 3. Both cases showed 50% increase in cell death compared to HAP monotherapy and a 7–13% increase compared to the simulation that mimicked an experimental schedule. Model parameter sensitivity analysis for the HAP+Vaso therapy is shown in Supplementary Fig. 4.

### Transient effect of pyruvate sensitizer

Another method to temporarily increase the extent of hypoxia within the tissue (and thus to enlarge the area of HAP activation), is to elevate the uptake of oxygen by the tumor cells. Experimental studies reported by Wojtkowiak et al.^13^ showed that Mia PaCa-2 cells exposed to exogenous *sodium pyruvate* increased the cells’ oxygen consumption rates in a dose-dependent manner. This study also showed that by combining a pre-treatment of *pyruvate* and *TH-302*, mice survival could be prolonged when compared to a mice cohort treated with *TH-302* alone.

Our mathematical model was parameterized with experimental data from^13^. The level of *pyruvate* in the vasculature was set up to ${\xi }_{0}=101{ag}/\mu {m}^{3}$ (after rescaling), and the time of bolus injection was set up to 25 minutes (Fig. 4a) to be comparable with the time of vasodilator activity, as we did not have information about the *pyruvate* plasma clearance time. Since *pyruvate* is a small molecule, we used the same diffusion coefficient as for oxygen: ${D}_{\gamma }={D}_{\xi }.$ The uptake of oxygen in *pyruvate*-exposed cells was amplified proportionally to the concentration of *pyruvate* (Fig. 2g in^13^). Three different sensitizer levels plus a control were tested in^13^, thus the cellular uptake of oxygen $\psi$ in our model is defined by Eq. (9) and in non-hypoxic areas depends on the level of the metabolic sensitizer $\xi$ with the amplifying factors: ${\psi }_{0}=1,{\psi }_{1}=5/3,{\psi }_{2}=7.5/3,{\psi }_{0}=12.5/3$ for the sensitizer levels of ${\xi }_{0}=9{ag}/\mu {m}^{3}$, ${\xi }_{1}=22{ag}/\mu {m}^{3}$, ${\xi }_{2}=88{ag}/\mu {m}^{3}$. With the addition of the sensitizer and its spread inside the tissue (Fig. 4b), the exposed cells consumed more oxygen, thus reducing oxygen dispersion and increasing the area of hypoxia (Fig. 4c). The minimum in tissue oxygenation was reached at around 40 minutes and then returned to its initial level within 2–2.5 hours. These levels were also quantified by tracing the normoxia-hypoxia border (Fig. 4d). We were able to simulate the induction of transient hypoxia within the tumor tissue by modulating oxygen uptake by the tumor cells as a function of metabolic sensitizer influx from the tumor vasculature.

### Optimizing HAP Activity Areas by Modulating Oxygen Uptake by Tumor Cells

**Fig. 5:**
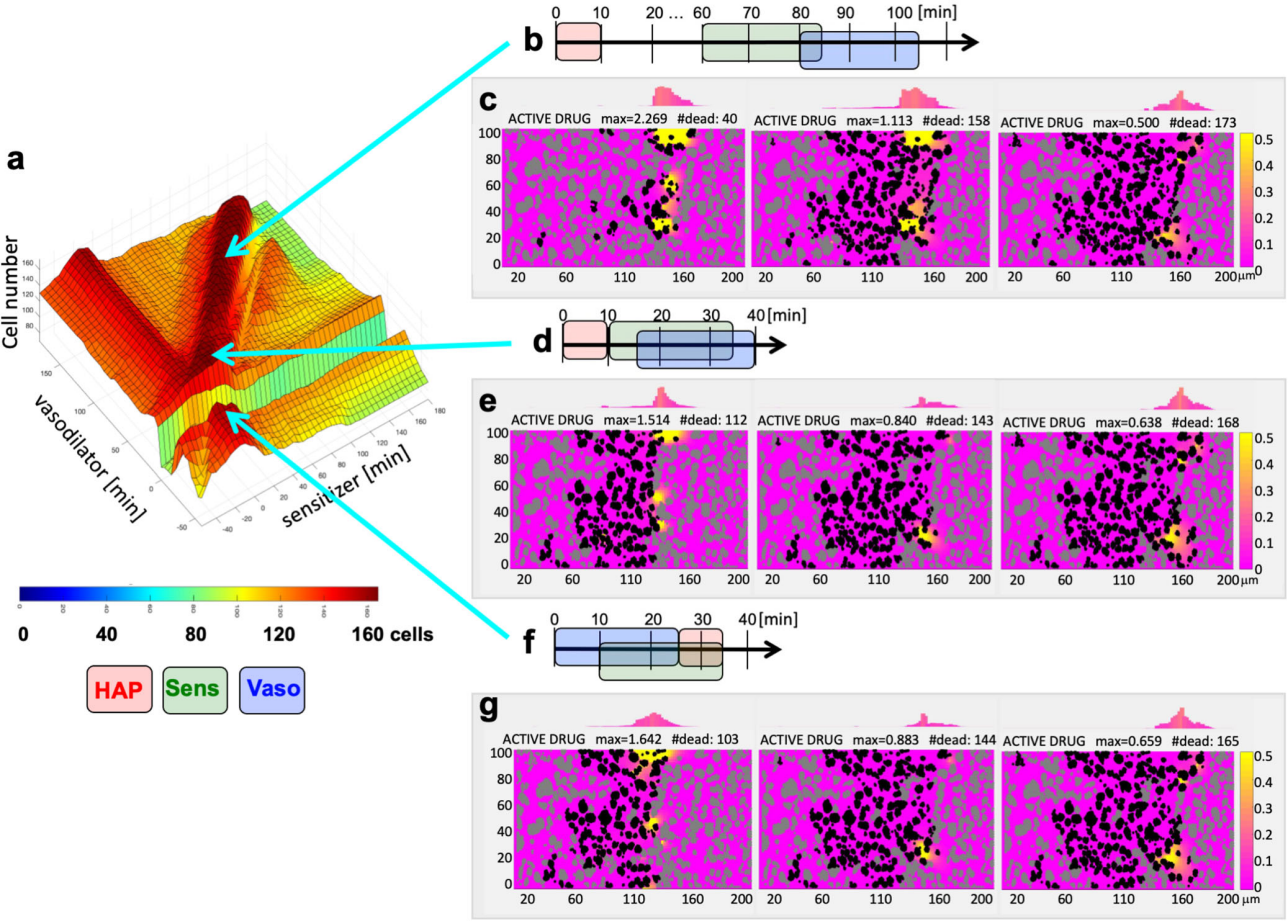
Optimizing schedules of HAP, vasodilator, and sensitizer combined. **a** A 3D parameter space showing the number of dead cells for the injection times of Sens and Vaso which are administered either before (− time) or after (+time) the HAP injection. Three local maxima are identified with the indicated treatment schedules: **b, d**, and **f**. Snapshots from each simulation (**c,** **e**, and **g**) showing dead cells at half an hour, at 1 hour, and at 3 hours, respectively.

Experimental studies reported by Wojtkowiak et al.^13^ showed that a combined therapy of *pyruvate* and *TH-302* reduced the growth of Mia PACa-2 tumors and prolonged mice life (Fig. 5b in^13^) when compared to *TH-302* alone. In these experiments, *pyruvate* was administered 30 minutes before *TH-302*^13^. We explored a wide range of sensitizer-HAP schedules in our study, with the goal of maximizing the number of dead cells. Fig. 4e shows the results of a series of computational simulations with a sensitizer administered either before (negative numbers on the x-axis) or after (positive numbers on the x-axis) the HAP. The vertical axis indicates the number of dead cells (out of 365) at 3 hours of the simulated time. These simulation results showed a wide plateau, which indicates that sensitizer submitted between 5 and 20 minutes before the HAP achieved similar effects, killing around 42.5% of cells (154–156 out of 365). The spatial distributions of the dead cells in two border cases are shown in Fig. 4f and Fig. 4g, respectively. For both schedules, HAP injection overlapped (either partially or entirely) with the sensitizer influx from the vasculature. This situation caused hypoxia to expand, initially due to increased cellular uptake; the subsequently injected inactive HAP encountered lower oxygen levels in larger areas, leading to increased zones of HAP activation. All schedules in the plateau showed 75% increase in cell death when compared to HAP monotherapy and a 6% increase compared to the simulation with experimental schedule. A more detailed set of snapshots from the most efficient schedule is presented in Supplementary Fig. 5. Model parameter sensitivity analysis for the HAP+Sens therapy is shown in Supplementary Fig. 4.

### Optimal scheduling of three-compound combination therapies

While simulations of HAP in combination with either a vasodilator or a sensitizer increased HAP efficacy, we still noted room for improvement. The combination of a treatment that increases cellular uptake of oxygen with a treatment that reduces vascular oxygen influx in the right order and at a right time could potentially elevate HAP effectiveness even more. We thus simulated schedules of three-compound combinations, in the numbers well beyond what would be possible to test in laboratory experiments. Figure 5a shows the 3D parameter space, with the number of dead cells in the z-axis and the time of sensitizer and vasodilator injections in the x- and y-axes, respectively. These times are defined relative to HAP injection; thus, the negative numbers denote injection before HAP, and positive numbers after HAP administration. Our simulations identified several distinct landscape patterns and determined local maxima that defined the optimal schedules for the combination of three compounds, as shown in Fig. 5.

The first pattern was a ditch-like area in Fig. 5a or a broad range of sensitizer injection times, but the vasodilator was injected around the same time as HAP. Since the vasodilator resulted in decreased blood flow in the vessel, it also reduced the influx of oxygen and any other intravascular compounds, such as HAP and/or sensitizer. The intratumoral concentration of HAP was thus smaller, and the total number of dead cells is diminished. When the HAP and sensitizer injection windows did not overlap completely with the time of vasodilator circulation in the blood system, the number of HAP-killed cells increased.

The second significant pattern was a ridge-like region located along the diagonal of the graph in Fig. 5a. In these schedules, the vasodilator was applied after the sensitizer, and both were administered after HAP injection. This approach allowed for the HAP to diffuse through the tumor tissue and be activated in the hypoxic areas before the sensitizer induced increase in oxygen uptake by the cells near the vasculature, thus enlarging the area of hypoxia and HAP activation. Finally, the injection of the vasodilator further increased the region of hypoxia by reducing oxygen influx. The global maximum in this case included an early injection of HAP, with a sensitizer administered 60 minutes after HAP, and a vasodilator 80 minutes after HAP (Fig. 5b). Three snapshots from this simulation, showing the extent of cell killing at 1/2, 1, and 3 hours of the simulated time, are shown in Fig. 5c. The average amounts of the active drug along the tissue are shown above each panel. The final count of the dead cells was 173 out of 365. For this scenario to work, the clearance of HAP from the tumor tissue must be slow (longer than 2 hours) to take advantage of the late injections of the enhancers. A more detailed set of snapshots from this simulation is shown in Supplementary Fig. 6. Analysis of robustness of model outcomes for this HAP+Sens+Vaso therapy is shown in Supplementary Fig. 7.

The second local maximum in this area appeared when HAP injection was followed in 10 minutes by the sensitizer, and the vasodilator 15 minutes after HAP (Fig. 5d). The three snapshots from this simulation shown in Fig. 5e indicate that the diffused HAP was activated much closer to the vasculature due to hypoxia induced by a double hit of the sensitizer and vasodilator in a short succession. The average amounts of the active drug along the tissue are shown above each panel. The final count of the dead cells was 168 out of 365, which is 3% less than in a global maximum case. A more detailed set of snapshots from this simulation is shown in Supplementary Fig. 8.

The final significant pattern was another ridge along the y-axis in Fig. 5a when the sensitizer was injected shortly before the HAP for the broad range of vasodilator injection times, except those described in the first pattern. In these cases, the prior injection of the sensitizer resulted in the expansion of hypoxia and HAP activation, and the hypoxia regions were enlarged once again when the vasodilator was administered. For many schedules in this pattern, the final number of dead cells was around 160, which was smaller than the other local optima we have discussed. Interestingly, the last local maximum in dead cell count was for the schedule in which the vasodilator was applied first, followed in 10 minutes by the sensitizer, and the HAP was administered 25 minutes after the vasodilator (Fig. 5f). In this case, the hypoxia was created first due to a vasodilator-induced lower oxygen influx and then sensitizer-induced oxygen uptake (although, the concentration of the sensitizer was lower due to vasodilation). Finally, the full-strength influx of HAP (just after the vasodilator was cleared from the vessel) resulted in its activation in the enlarged hypoxic area. The three corresponding snapshots from this simulation are shown in Fig. 5g, and the average amounts of the active drug along the tissue are shown above each panel. The final dead cell count was 165 out of 365, which was 5% less than in a global maximum case. A more detailed set of snapshots from this simulation is shown in Supplementary Fig. 9.

## Discussion

Since chronic hypoxia is among the characteristics of solid tumors, therapies targeted towards hypoxic cells are being developed and tested in clinical trials both as monotherapy and in combination with other treatments. The behavior of hypoxia-activated pro-drugs is complex and when combined with compounds that can also modify tumor microenvironment, predicting what treatment protocols will be most effective can be difficult. In this study, we extended our previously developed model of micro-pharmacology^13,19,20^ to include the scheduling of two- and three-drug combinations with the goal of maximizing tumor cell elimination. In this model, we used tumor histology images as a simulations domain and were inspired by in vivo experiments in which HAPs (*TH-302*) were combined either with a vasodilator (*hydralazine*) that decreased the influx of oxygen or with a metabolic sensitizer (*pyruvate*) that increased cellular uptake by tumor cells. Our simulations showed that for the combination of HAP with the vasodilator, the distribution of scheduling times was bimodal, with one local maximum when the vasodilator was given before HAP and one local maximum, when the vasodilator was given after HAP. For each of these local maxima, the number of dead cells was about 42% and 53% higher, respectively, than for the HAP monotherapy. The combination of HAP with the sensitizer showed a 15 min-long plateau in scheduling times, when all simulations were equally successful in killing tumor cells, and resulted in a 77–79.5% increase in the number of dead cells when compared to HAP administered alone. The carefully scheduled three-compound treatments, however, resulted in doubling the number of dead cells in comparison to HAP monotherapy; the schedule in which active HAP clearance was assumed to be long-lasting resulted in a global maximum of nearly 95% increase (173 cells vs. 87 cells for HAP monotherapy). However, it is worth to notice, that even in this most successful scenario, only about 47.5% of cells in the tissue were dead. More cells might still be killed if the simulations are run beyond the 3 hours considered in this study, as the area close to the right end of our computational tissue was hypoxic, and some amount of active HAP still remained in this area. But the region closer to the left edge of the domain, where the vessel is located, is not likely to experience more HAP-related deaths, as this area is always above the hypoxia level. Those regions and cells must be targeted with other therapies, such as classical cytotoxic or anti-mitotic chemotherapies. While we used experimental data to develop our mathematical model, the simulated results have not been validated using in vivo tumors. It would be especially interesting to test which of the predicted optimal schedules for three-compound combination therapies are confirmed by mice studies, since there is no a priori knowledge of whether the clearance of *TH-302* from the tissue interstitium is fast or is a long-lasting process. Such experimental studies would also verify whether computational methods, such as the one presented here, should be routinely incorporated in designing in vivo laboratory experiments to optimize multi-drug treatment schedules.

Further improvements in our mathematical model may be considered. We previously showed that an active HAP could experience the bystander effect: that is, the active drug may be observed in the non-hypoxic areas, when it should not have been activated. In our previous work, this effect was only possible when the diffusion coefficient of active HAP was increased to that comparable to oxygen^19^. As a result, the already-activated HAP diffused to the well-oxygenated areas. A similar effect may be obtained for smaller velocities of the interstitial fluid. The one we used in the present study was based on velocities observed in laboratory experiments^23^, although these were the average values. Smaller velocities would lower the advection effect and the Péclet number, and, as a result, the compound transport would be diffusion-dominated (as we showed previously in^20^). This approach, in turn, could result in active HAP presence in the oxygenated areas. Our current model does not directly incorporate cell metabolic processes. As a result, the enhancement of oxygen consumption in cells exposed to *pyruvate* is immediate. This may not be biologically accurate, as alterations in cell metabolism may take some time to occur. Our future research could address this issue by modeling cell metabolism in more detail. We could also implement multiple administrations of HAP, the vasodilator, and the sensitizer to test whether cells near the vasculature would be affected by active HAP. Moreover, the histology tissue used in the present study as a computational domain has a quite simple geometry, with only one vessel located along the left domain boundary. We intentionally chose this geometry to reduce topological complexity, since the drug and oxygen kinetics already generated a complex microenvironment. In future research, in order to perform simulations on more realistic tissue architectures, we could use more complex tissues with multiple vessels (as we did in^25^), vessels with different cross-sections and shapes, branched structures, and vessels with non-uniform oxygen content. Moreover, our computational model can be extended to a 3D space, as all mathematical equations for 3D calculations are already available. This includes a 3D cut-off function for an exact solution of the regularized Stokeslets method^26,27^, and constructing the 3D non-penetration stencil for implementation of the diffusion equation that considers 6 neighboring sides instead of 4, as in 2D implementation. It is also possible to use the 3D irregular compact surfaces to represent cell shapes and cylindrical surfaces to represent vessels, since our methods use only coordinates of boundary points from the discretized surfaces. In principle, the 3D cells and vessels can be segmented from the co-registered histology images of consecutive tissue slices (z-stacks)^28^.

Although we utilized histology from mouse tumors as a base for all model simulations, it is also possible to use images of patients’ tumors from biopsies routinely collected in the clinic. Moreover, while our model was based on *TH-302* data in pancreatic tumors, the methods we have developed can be applied to other hypoxia activated pro-drugs and other solid tumors. As with every new application, the model will need to be calibrated with this new data. Clinical tissue processing (cutting slices, staining, image scanning, cell and vessel segmentation) can be done in a manner similar to that used with mouse data. So far, we have applied our methods to tissues of areas up to several square millimeters^19,25^. For larger tissues, such as human tumors, it may be necessary to develop methods that will stitch several smaller tissue subregions as it is currently done in advanced image analyses. For modeling *TH-302*, there is already information available on its administration in clinical trials for pancreatic and other tumors^29–31^. However, data for treatment combinations will be available only if the compounds (other drugs, vasodilators, metabolic sensitizers) are already approved for clinical use by health agencies. Some additional in vitro experiments will need to be done to assess uptake rates of the involved compounds by the given tumor cells. While all described data collections are technically possible, this procedure will require the initiation of a clinical trial. The more feasible application of our techniques, and our future goal, is to use this methodology as a decision-support tool and, as such, to apply the techniques only to the data that are already collected for the use of pathologists. This tool could provide clinicians with additional information about the pharmacologic or metabolic status of a tumor based on that tissue histology slice.

Our current work on hypoxia-activated pro-drugs differs from previously published papers on this topic in terms of both mathematical formulation and applications. Lindsay et al.^32^ simulated therapeutic strategies that combine *evofosfamide (TH-302)* with a standard therapy of a tyrosine kinase inhibitor (*erlotinib*) to treat non-small cell lung cancer. The authors developed a stochastic mathematical model to optimize treatment strategies that would delay the emergence of drug resistance vs. those that reduce tumor burden. Meaney et al.^33^ investigated how a combination of *TH-302* and anti-angiogenic therapy AA, (*combretastatin*), either administered directly or in the form of nanocell delivery, could improve treatment efficacy in glioblastomas. The authors used a system of partial differential equations (PDEs) and showed that AA/HAP combination therapy was more effective when administered through nanocells. Hamis et al.^34^ used an on-lattice cellular automata mathematical model to investigate a combination of *TH-302* with radiotherapy in the multicellular spheroids of human chondrosarcoma. The authors showed that HAP could function as an intensifier for the ionizing radiation and that a proper scheduling of HAP and radiation could affect treatment efficacy. The interplay between radiation and HAP (*SN30000*) in colon cancer multicellular spheroids was simulated by Mao et al.^35^. The authors developed a hybrid cellular automata model to mimic the spheroid architecture and used it to predict clonogenic cell killing in spheroids. Foehrenbacher et al.^36^ used a spatial pharmacokinetic/pharmacodynamic model to address the bystander effect of HAP (*PR-104A*) in squamous cell carcinoma and in colon cancer. The authors showed via simulations that the bystander effect contributed 30% and 50% of HAP activity in these tumors, respectively. Our model was based on *TH-302* experiments, similarly to some of the models mentioned above, although, we investigated scheduling for a combination of HAP with two enhancers that transiently increased the extent of hypoxia, but did not kill the tumor cells directly. We also used the fluid-structure interaction methods to model interstitial fluid and compound advection. These methods were not considered in the previously published papers.

In summary, our study indicates that the three-compound therapy administered in the defined order with the well-planned injection times will improve cancer response. Designing optimal schedules, that is, determining the right drug sequence, injection timing, and dosing for combinations of multiple compounds, is beyond what is feasible with empirical testing. Especially, if the administered drugs also modify the tumor microenvironment. This requires methods that can inspect large numbers of possible schedules relatively fast and inexpensively, such as mathematical modeling, optimization techniques, and computational simulations. The treatment schedule optimization research is an excellent example of the benefits that mathematical modeling and simulations can bring to the biological and medical sciences.

## Methods

Our general methodology is based on our deterministic micro-pharmacology model^13,19,20^ and combines the fluid-structure interactions method of regularized Stokeslets^27^ with reaction-advection-diffusion equations. The method consists of the following steps: (i) design an in silico tissue sample as a computational domain for simulations; (ii) compute forces that result in the desired fluid and oxygen/drug supply from the blood capillaries; (iii) compute the interstitial fluid velocity field driven by these forces; (iv) compute compound kinetics (diffusivity, advection, decay, and conversion to an active form); and (v) compute the cellular uptake of oxygen and active HAP, and update the cell phenotype as needed. Finally, iteratively repeat these steps to advance the penetration of all compounds through the tumor tissue and the tumor tissue response.

### Virtual tumor tissue

**Fig. 6:**
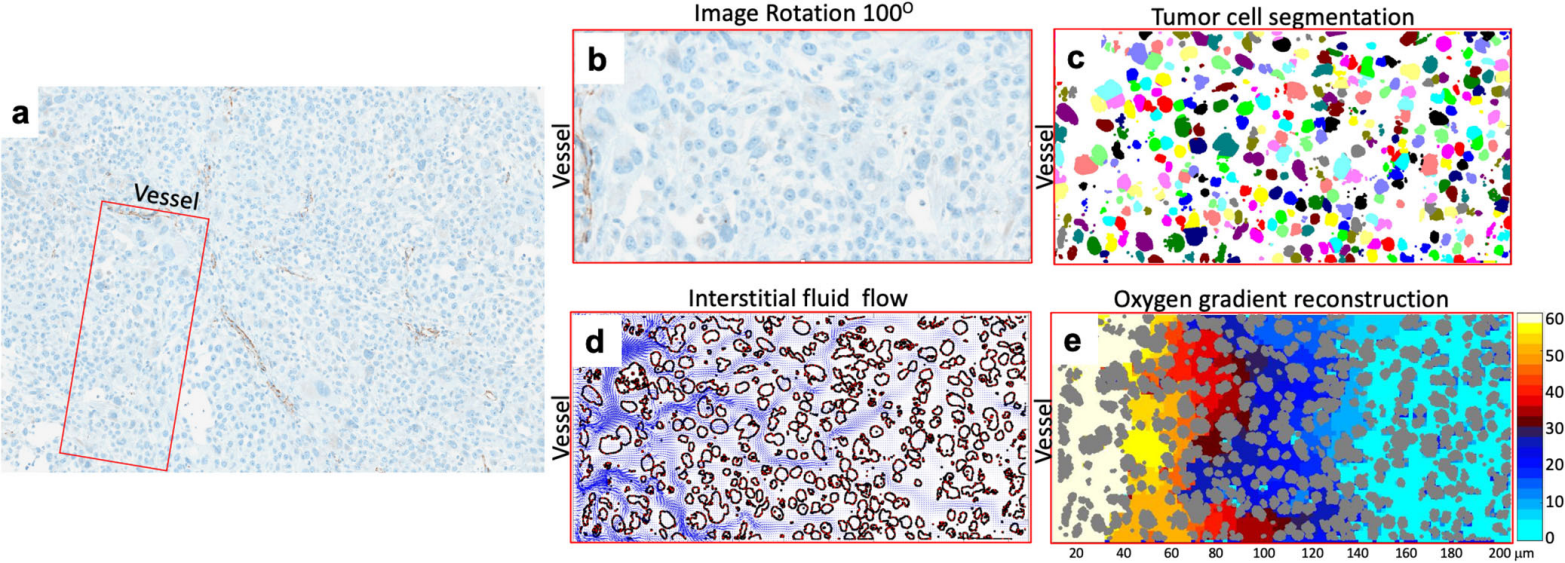
Design of a digitized tumor tissue. **a** An image of the slice of Mia PaCa-2 tumor tissue with the selected region of interest (ROI) indicated by the red box. **b** A patch of tumor tissue with a vessel located along the left domain boundary (ROI from **a**.). **c** The segmented tumor cells from **b**. **d** The interstitial fluid flow pattern in the extracellular space between the segmented cells from **c**., calculated using the regularized Stokeslet method. **e** A stable gradient of oxygen in the extracellular space between the segmented cells from **c**., calculated using the reaction-diffusion-advection PDEs (including the fluid flow from **d**.).

The virtual tumor tissue was created using a histology image from a mice xenograft of Mia PaCa-2 pancreatic tumor^13^. A slice of untreated tumor was stained with hematoxylin and eosin (H&E) markers to identify tumor cells and with CD-31 immunohistochemical marker to localize tumor vasculature (Fig. 6a). The scanned digital image was then used to select a small patch of tissue that contained a vessel along the image edge (Fig. 6b). This image formed the basis for creating a two-dimensional (2D) computational domain $\left(\Omega =\left[{x}_{\min },{x}_{\max }\right]\times [{y}_{\min },{y}_{\max }]\right)$ for all model simulations. The individual tumor cells ${\left\{{\Gamma }_{l}\right\}}_{l=1}^{N}$ were captured using the *ImageJ* (NIH) software^37^ and segmented using an in-house algorithm based on the K-means clustering method (Fig. 6c). The extracellular space outside the cells $\left(\bar{\Omega }=\Omega {\setminus}\mathop{\sum }_{l=1}^{N}{\Gamma }_{l}\right)$ was used for the interstitial fluid, metabolites and drugs transport. The flow pattern of the interstitial fluid (Fig. 6d) resulted from vascular influx along the left domain boundary and passage past the obstacles formed by all cell boundaries $\left(\partial \Gamma ={\sum }_{l=1}^{N}\partial {\Gamma }_{l}\right).$ This fluid flow was calculated using the regularized Stokeslets method that is time-invariant, since all cells are assumed to be non-motile and non-proliferative due to the short time frame (a couple of hours) considered here. All simulations started with a stable distribution of oxygen (Fig. 6e), which arose as a balance between constant vascular supply and cellular absorption, and was calculated using the reaction-diffusion-advection equation.

### Tumor cells

Each tumor cell $\left({\Gamma }_{l}\right)$ was characterized by its individual shape and area $\left({{\rm{{\rm A}}}}_{l}\right)$, as well as cell’s boundary points with coordinates ${\left\{{{\boldsymbol{X}}}_{i}^{l}=\left({X}_{i}^{l},{Y}_{i}^{l}\right)\right\}}_{i=1\ldots {N}_{l}}$. These points played the role of cell pseudo-receptors used as sites of oxygen and active drug absorption, and as obstacles to the interstitial fluid flow (Fig. 6d). The number of cell boundary points $\left({{\rm{N}}}_{l}\right)$ depended on cell size and thus varied between individual cells.

### Interstitial fluid

The interstitium was modeled as a homogeneous gel representing the extracellular matrix (ECM) interpenetrated by the interstitial fluid. Since the characteristic cell-tissue length scale $L$ is on the order of microns and the characteristic interstitial fluid velocity speed $U$ is on the order of microns per second, the corresponding Reynolds number ($\mathrm{Re}=\rho {LU}/\mu$, where $\rho$ is fluid density and $\mu$ is fluid viscosity) was small, and the interstitial fluid flow could be approximated by the Stokes equations Eqs. (1–2):

1 $$\mu \,\varDelta {\bf{u}}({\bf{x}})=\nabla p({\bf{x}})-{\bf{f}}({\bf{x}})$$

$${\rm{and}}$$

2 $$\nabla \cdot {\bf{u}}({\bf{x}})=0,$$

where, ${\bf{x}}$ is an arbitrary point in the domain $\Omega$, $p$ is the fluid pressure, ${\bf{u}}$ is the fluid velocity field, and the external force ${\bf{f}}$ comprises of: ${{\bf{f}}}_{{in}}$ along the vessel to generate the interstitial influx, ${{\bf{f}}}_{{bnd}}$ along the top and bottom domain boundaries ($\partial \Omega$) to define the no-slip condition, and ${{\bf{f}}}_{{cell}}$ along the boundaries of all cells ($\partial \Gamma$) to keep them immobile:

3 $${\bf{f}}\left({\bf{x}}\right)=\left\{\begin{array}{l}\begin{array}{l}{{\bf{f}}}_{{in}}\left({\bf{x}}\right)+{{\bf{f}}}_{{bnd}}({\bf{x}}) \qquad\qquad{\rm{if}}\quad{\bf{x}}\in \partial \Omega \end{array}\\ \begin{array}{l}{{\bf{f}}}_{{cell}}({\bf{x}}) \qquad\qquad\quad\qquad{\rm{if}}\quad{\bf{x}}\in \partial \Gamma \end{array}\\ \begin{array}{l}0 \qquad\qquad\qquad\qquad\qquad{\rm{otherwise}}.\end{array}\end{array}\right.$$

This method creates the physiologically relevant interstitial fluid flow. Equations Eqs. (1)-(3) are solved using the classical fluid-structure interaction method of regularized Stokeslets^27,38^, in which each force concentrated at a point ${{\bf{x}}}_{0}$ is smoothed over a disk of radius $\varepsilon$ using the cut-off function ${\varphi }_{\varepsilon }$, Eqs. (4)-(5):

4 $${\bf{f}}\left({\bf{x}}\right)={{\bf{f}}}_{o}{\varphi }_{\varepsilon }\left({\bf{x}}-{{\bf{x}}}_{{\bf{0}}}\right)$$

and

5 $${\varphi }_{{\varepsilon }}({\bf{x}})=\frac{2{{\varepsilon }}^{4}}{\pi {(||{\bf{x}}|{|}^{2}+{{\varepsilon }}^{2})}^{3}}.$$

The contribution from finitely many discrete forces ${{\bf{f}}}_{k}$ defined on the cell and domain boundary points ${{\bf{x}}}_{k}$ yields the following exact expression for the fluid velocity ${\bf{u}}$ at the arbitrary point ${\bf{x}}\in \Omega$, Eq. (6):

6 $$\displaystyle{\bf{u}}\left({\bf{x}}\right)=\dfrac{1}{4\pi \mu }\mathop{\sum }\limits_{k=1}^{M}\left\{\left(\frac{2{\varepsilon }^{2}}{{r}_{k}^{2}+{\varepsilon }^{2}}-{\mathrm{ln}}\left({r}_{k}^{2}+{\varepsilon }^{2}\right)\right){{\boldsymbol{f}}}_{k}+\frac{1}{{r}_{k}^{2}+{\varepsilon }^{2}}\left[{{\bf{f}}}_{k}\,\cdot \,\left({\bf{x}}-{{\bf{x}}}_{k}\right)\right]\left({\bf{x}}-{{\bf{x}}}_{k}\right)\right\}$$

where $M$ is the total number of forces and *r*_*k*_ = ||**x** − **x**_*k*_||$.$ The regularization parameter $\varepsilon$ was equal to the distance between cell boundary points to minimize numerical errors^38^.

Equation Eq. (6) can be inverted to calculate forces that must be applied at points ${{\bf{x}}}_{k}$ to achieve the desired velocity ${\bf{u}}({{\bf{x}}}_{k})$^27^. Thus for the imposed influx velocity (${\bf{u}}\left({{\rm{x}}}_{\min },y\right)={{\bf{u}}}_{{\boldsymbol{in}}}$ for ${y}_{\min }\le y\le {y}_{\max }$), and boundary velocities ${\boldsymbol{(}}{{\bf{u}}}_{{\boldsymbol{cell}}}\,{\boldsymbol{=}}\,{\bf{u}}\left({{\bf{x}}}_{\partial \Gamma }\right)=0{\boldsymbol{)}}$ and ${\boldsymbol{(}}{{\bf{u}}}_{{\boldsymbol{bnd}}}\,{\boldsymbol{=}}\,{\bf{u}}\left({{\bf{x}}}_{\partial \Omega }\right)=0{\boldsymbol{)}}$, we use the generalized minimal residual method (*GMRES*) to calculate the corresponding forces ${{\boldsymbol{(}}{\bf{f}}}_{{\boldsymbol{in}}}{\boldsymbol{,}}{{\bf{f}}}_{{\boldsymbol{cell}}}{\boldsymbol{,}}{{\bf{f}}}_{{\boldsymbol{bnd}}}{\boldsymbol{)}}$ at these points. These forces are then used again in Eq. (6) to calculate the interstitial fluid velocity field in the whole domain, as shown in Fig. 6d. The fluid velocity field remains fixed in all simulations except when the vasodilator is administered, where the fluid flow magnitude but not the direction is changed. This fluid flow was then used in the kinetic equations for oxygen and all other compounds discussed below.

### Oxygen spatio-temporal dynamics

Oxygen is supplied from a vessel located along the left boundary of the domain, is carried through the interstitial space via a combination of advective and diffusive transports, and is absorbed at the cell boundaries. The spatio-temporal changes in oxygen concentration $\gamma$ are described by the following reaction-diffusion-advection equation Eq. (7):

7 $$\frac{\partial \gamma ({\bf{x}},\,t)}{\partial t}=\mathop{\underbrace{{D}_{\gamma }\varDelta \gamma ({\bf{x}},\,t)}}\limits_{diffusion}+\mathop{\underbrace{{\bf{u}}({\bf{x}})\cdot \nabla \gamma ({\bf{x}},\,t)}}\limits_{advection}+\mathop{\underbrace{\psi (\xi ({\bf{x}},\,{t}),\,\gamma ({\bf{x}},\,t)){\sum }_{l=1}^{N}{\sum }_{i=1}^{{N}_{l}}{\chi }_{{\varepsilon }}({\bf{x}},\,{{\bf{X}}}_{i}^{l})}}\limits_{cellular\,uptake}$$

where ${D}_{\gamma }$ is the effective diffusion coefficient, ${\bf{u}}$ is the interstitial fluid velocity, and $\psi$ is the uptake function. The indicator function ${\chi }_{\varepsilon }\left({\bf{x}},{{\bf{X}}}_{i}^{l}\right)$ is used to define the transfer of information between the Cartesian grid ${\bf{x}}$ and the Lagrangian coordinates ${{\bf{X}}}_{i}^{l}$ of cell pseudo-receptors$.$ The indicator function is defined as follows:

8 $${\chi }_{{\varepsilon }}({\bf{x}},\,{\bf{X}})=\left\{\begin{array}{cc}1 & {\rm{if}}\,\,||{\bf{x}}-{\bf{X}}||\, < \,{\varepsilon }\\ 0 & {\rm{otherwise}}.\end{array}\right.$$

The areas with oxygen below the threshold value ${\gamma }_{{hyp}}$ are hypoxic. For cells located in the regions of severe hypoxia ($\gamma \left({\bf{x}},t\right)\, < \,0.5{\gamma }_{{hyp}}$), their oxygen uptake rate $\psi$ is proportional to oxygen concentration, while in the regions with higher oxygen content, the cellular uptake of oxygen is constant. This uptake rate was computationally optimized to achieve the tissue normoxia-hypoxia border (as described in details in the Result section) at $130\mu m$ from the vessel. However, following experiments with the metabolic sensitizer *pyruvate* (^13^ and the Result section), the baseline uptake rate of oxygen is amplified proportionally to the concentration of sensitizer $\xi$ with the proportionality factor ${\psi }_{k}$ (for $k=0,\ldots ,3$, since experiments in^13^ tested 4 different concentrations of $\xi$). The cellular uptake of oxygen thus depends on concentrations of oxygen and metabolic sensitizer, and is defined in Eq. (9):

9 $$\psi (\xi ({\bf{x}},\,{t}),\gamma ({\boldsymbol{x}},t))=\left\{\begin{array}{cl}{\gamma }_{0}\gamma ({\bf{x}},\,t) & {\rm{if}}\,\gamma ({\bf{x}},\,t)\, < \,0.5\,{\gamma }_{hyp}\\ {\gamma }_{0} & {\rm{if}}\,\gamma ({\bf{x}},\,t)\ge 0.5\,{\gamma }_{hyp}\,{\rm{and}}\,\xi ({\bf{x}},t)=0\\ {\gamma }_{0}{\psi }_{0} & {\rm{if}}\,\gamma ({\bf{x}},\,t)\ge 0.5\,{\gamma }_{hyp}\,{\rm{and}}\,0\le \xi ({\bf{x}},t)\,<\,{\xi }_{0}\\ {\gamma }_{0}{\psi }_{1} & {\rm{if}}\,\gamma ({\bf{x}},\,t)\ge 0.5\,{\gamma }_{hyp}\,{\rm{and}}\,{\xi }_{0}\le \xi ({\bf{x}},t)\,<\,{\xi }_{1}\\ {\gamma }_{0}{\psi }_{2} & {\rm{if}}\,\gamma ({\bf{x}},\,t)\ge 0.5\,{\gamma }_{hyp}\,{\rm{and}}\,{\xi }_{1}\le \xi ({\bf{x}},t)\,<\,{\xi }_{2}\\ {\gamma }_{0}{\psi }_{3} & {\rm{if}}\,\gamma ({\bf{x}},\,t)\ge 0.5\,{\gamma }_{hyp}\,{\rm{and}}\,{\xi }_{2}\le \xi ({\bf{x}},t)\end{array}\right.$$

The zero-velocity values imposed on cell boundaries guarantee that oxygen (and drugs) carried via advective transport cannot cross the cell boundaries accidentally. For the diffusive transport, we modified the classical numerical 4-stencil implementation by omitting stencil points inside the cells (Supplementary Methods). This will assure that the cell interior cannot be penetrated by oxygen unless it is directly absorbed by the cell.

The oxygen boundary conditions consist of the constant influx $\gamma \left({{\rm{x}}}_{\min },y,t\right)={\gamma }^{{in}}$ along the left domain boundary (for ${y}_{\min }\le y\le {y}_{\max }$), no-flux conditions along the bottom and top boundaries, and zero concentration along the right domain boundary. The initial condition for oxygen is shown in Fig. 6e. This is a numerically stabile gradient, ${\gamma }^{n}\left({\bf{x}}\right)$, satisfying: ${\Vert {\gamma }^{n}({\bf{x}})-{\gamma }^{n-1}({\bf{x}})\Vert }_{2}/({N}_{i}\cdot {N}_{j})\, < \,{10}^{-10}$, which is calculated for oxygen distributions from two consecutive iterations and normalized by the number of computational grid points.

### Drug spatio-temporal dynamics

The hypoxia-activated pro-drug is supplied in an inactive form from the vessel located along the left boundary of the domain and is carried through the interstitial space via a combination of advective and diffusive transports. The inactive drug is not absorbed by the cells until it is converted to its active form in the hypoxic regions of the tissue. The active drug is also carried via advective and diffusive transports, is subject to natural decay, and is absorbed by the cells through their pseudo-receptors. The spatio-temporal changes in concentrations of both an inactive pro-drug ${\eta }_{i}$ and its activated form ${\eta }_{a}$ are described by the following equations Eqs. (10, 11):

10 $$\frac{\partial {\eta }_{i}({\bf{x}},\,t)}{\partial t}=\underbrace{{D}_{{\eta }_{i}}\varDelta {\eta }_{i}({\bf{x}},\,t)}_{diffusion}+\underbrace{{\bf{u}}({\bf{x}})\cdot \nabla {\eta }_{i}({\bf{x}},\,t)}_{advection}-\underbrace{\varphi (\gamma ({\bf{x}},\,{t})){\eta}_{i} ({\bf{x}},\,t)}_{activation}$$

11 $$\begin{array}{c}\dfrac{\partial {\eta }_{a}({\bf{x}},\,t)}{\partial t}=\underbrace{{D}_{{\eta }_{a}}\varDelta {\eta }_{a}({\bf{x}},\,t)}_{diffusion}+\underbrace{{\bf{u}}({\bf{x}})\cdot \nabla {\eta }_{a}({\bf{x}},\,t)}_{advection}+\underbrace{\varphi (\gamma ({\bf{x}},\,{t})){\eta }_{i}({\bf{x}},\,t)}_{activation}\\-\, \underbrace{\alpha {\eta }_{a}({\bf{x}},\,{t})\mathop{\sum }\limits_{l=1}^{N}\mathop{\sum }\limits_{i=1}^{N}{\chi }_{{\varepsilon }}({\bf{x}},\,{{\bf{X}}}_{i}^{l})}_{cellular\,uptake}-\underbrace{{\omega }_{a}{\eta }_{a}({\bf{x}},\,t)}_{decay}\end{array}$$

where ${D}_{{\eta }_{i}}$ and ${D}_{{\eta }_{a}}$ are the diffusion coefficients for the inactive and active drugs, respectively; ${\bf{u}}$ is the interstitial fluid velocity, $\alpha$ is the rate of cellular uptake of the active drug, and ${\omega }_{a}$ is the active drug decay rate. The indicator function ${\chi }_{\varepsilon }\left({\bf{x}},{{\bf{X}}}_{i}^{l}\right)$ is defined in Eq. (8). The inactive drug is converted into its active form at a rate ${\varphi }^{{conv}}$ only if the oxygen level is below the hypoxic threshold ${\gamma }_{{hyp}}$, as defined in Eq. (12):

12 $$\varphi \left(\gamma \left({\bf{x}},t\right)\right)=\left\{\begin{array}{l}\begin{array}{cc}{\varphi }^{{conv}} & {\rm{if}}\,\,\,\gamma \left({\bf{x}},t\right)\le {\gamma }_{{hyp}}\end{array}\\ \begin{array}{l}0 \qquad\quad{\rm{otherwise}}\end{array}\end{array}\right.$$

The drug boundary conditions include the influx of the inactive drug ${\eta }_{i}\left({{\rm{x}}}_{\min },y,t\right)={\eta }_{i}^{{in}}$, and a zero value for the activated drug ${\eta }_{a}\left({{\rm{x}}}_{\min },y,t\right)=0$ (in both cases ${y}_{\min }\le y\le {y}_{\max }$). No-flux conditions are imposed along the bottom and top boundaries, and zero concentration is set along the right domain boundary for both inactive and active drugs. Initial concentrations of both drugs are uniformly equal to zero: ${\eta }_{i}\left({\bf{x}},{t}_{0}\right)={\eta }_{a}\left({\bf{x}},{t}_{0}\right)=0$.

### Cell death

Each cell ${\Gamma }_{l}$ can absorb the active drug from its immediate vicinity, and drug accumulation ${\Gamma }_{l}^{\eta }$ is described by the ordinary differential equation Eq. (13):

13 $$\frac{\partial {\varGamma }_{l}^{\eta }(t)}{\partial t}=\mathop{\underbrace{{\sum }_{{{\bf{X}}}_{i}^{l}\in \partial {\varGamma }_{l}}{\sum }_{{\bf{x}}\in \varOmega }\alpha \,{\eta }_{a}({\bf{x}},\,{t})\,{\chi }_{{\varepsilon }}({\bf{x}},\,{{\bf{X}}}_{i}^{l}),}}\limits_{cellular\,uptake}$$

where $\alpha$ is the uptake rate, the summations are taken over all cell pseudo-receptors and all grid points ${\bf{x}}$ in cell’s receptor ${{\bf{X}}}_{{\boldsymbol{i}}}^{{\boldsymbol{l}}}$ neighborhood as defined by the indicator function. Cell death is triggered when the average concentration of the drug accumulated in that cell (normalized by the cell area, ${\varGamma }_{l}^{\eta }/\overline{\overline{{\varGamma }_{l}}}$) exceeds the drug’s lethal threshold ${\eta }_{a}^{{dead}}$. In such cases, the cell stops all its absorption processes, but its remnants remain in the tissue, since we assume no clearing processes over the simulated 3-hour time frame. The number of dead cells ${N}_{{dead}}$ is used to compare effectiveness of the proposed optimal schedules.

### Sensitizer spatio-temporal dynamics

The sensitizer is supplied from the vessel and is carried through the interstitial space via a combination of advective and diffusive transports. The spatio-temporal changes in sensitizer $\xi$ concentration are described by equation Eq. (14):

14 $$\frac{\partial \xi ({\bf{x}},\,{t})}{\partial t}=\underbrace{{D}_{\xi }\varDelta \xi ({\bf{x}},\,{t})}_{{diffusion}}+\underbrace{{\bf{u}}({\bf{x}})\cdot \nabla \xi ({\bf{x}},\,{t})}_{{advection}}$$

where ${D}_{\xi }$ is the sensitizer diffusion coefficient and ${\bf{u}}$ is the velocity of the interstitial fluid. The goal of administering the sensitizer $\xi$ is to increase the uptake of oxygen $\gamma$ by the tumor cells exposed to $\xi$ in a dose-dependent way described in Eq. (9). The boundary conditions include the influx of the sensitizer from a vessel: $\xi \left({{\rm{x}}}_{\min },y,t\right)={\xi }^{{in}}$ (${y}_{\min }\le y\le {y}_{\max }$), no-flux conditions along the bottom and top boundaries, and zero concentration along the right domain boundary. Initially, there was no sensitizer in the whole domain: $\xi \left({\bf{x}},{t}_{0}\right)=0$.

### Vasodilator spatio-temporal dynamics

The goal of intravenous administration of the vasodilator $\phi$ is to decrease the blood flow in tumor vasculature, which also results in the decreased influx of oxygen and all other blood-borne compounds (inactive drug and sensitizer) during the time the vasodilator is present in blood circulation ($\phi =1$). Thus, we model the effects of the vasodilator by modifying the vessel boundary condition without explicitly modeling the concentration of the vasodilator. This scenario is described by Eq. (15), where $\lambda$ defines the rate of decrease of the influx of the interstitial fluid flow ${{\bf{u}}}_{{in}}$, the oxygen influx ${{\rm{\gamma }}}^{{in}}$, the inactive drug influx ${{\rm{\eta }}}_{i}^{{in}}$, and the sensitizer influx ${{\rm{\xi }}}^{{in}}$, if they are present in the circulation at any time during the period the vasodilator is present $\left[{t}_{1},{t}_{2}\right]$:

15 $$\left\{\begin{array}{l}\begin{array}{l}{\bf{u}}\left({\bf{x}}\right)={\lambda }{{\bf{u}}}_{in}\qquad\qquad\,\,\,\,\,\,{\rm{for}}\qquad{{t}}_{{1}}\le {t}\le {{t}}_{{2}}\,\,\,\,{\rm{when}}\,\,\,\,{\phi}={1}\\ {\gamma }\left({{x}}_{{min}},{y},{t}\right)={\lambda}{{\gamma }}^{{in}}\qquad{\rm{for}}\qquad{{t}}_{{1}}\le {t}\le {{t}}_{{2}}\,\,\,\,{\rm{when}}\,\,\,\,{\phi}={1}\end{array}\\ \begin{array}{l}{{\eta}}_{{i}}\left({{x}}_{{min}},{y},{t}\right)={\lambda}{{\eta}}_{{i}}^{{in}}\quad\;\;\,{\rm{for}}\qquad{{t}}_{{1}}\le {t}\le {{t}}_{{2}}\,\,\,\,{\rm{when}}\,\,\,\,{\phi}={1}\\ {\xi}\left({{x}}_{{min}},{y},{t}\right)={\lambda}{{\xi}}^{{in}}\qquad\,{\rm{for}}\qquad{{t}}_{{1}}\le {t}\le {{t}}_{{2}}\,\,\,\,{\rm{when}}\,\,\,\,{\phi}={1}\end{array}\end{array}\right.$$

All model parameter values are listed in Table 1. More information on model implementation can be found in Supplementary Methods.

**Table 1:** Model physical and computational parameters.

Parameter	Symbol	Value	References
Domain	$\Omega$	[0,200]*x*[0,100] *μm*^2^	Histology, Fig. 6
Time step	$\triangle t$	1.5 × 10^−3^ *min*	num. stability
Grid width	$\triangle x=\triangle y=h$	2 *μm*	num. stability
Regularization parameter	${\rm{\varepsilon }}$	2 *μm*	^38^
Final time	${t}^{{end}}$	3 *hours*	
Interstitial fluid viscosity	$$\mu$$	0.04 *mg/*$$($$*μm∙min*$$)$$	water at 38 ^o^C
Interstitial fluid influx velocity	${{\boldsymbol{u}}}_{{in}}$	$1$ *μm/s*	^22,23^
Velocities at cell and domain boundaries	${{\boldsymbol{u}}}_{{cell}}={{\boldsymbol{u}}}_{{bnd}}$	$0$ *μm/s*	immobile cells
Oxygen diffusion coefficient	*D*_*γ*_	10^3^ *μm*^2^⁄*min*	^39,40^
Hypoxia threshold	${\gamma }_{{hypo}}$	$10$ *mmHg*	^1,24^
Oxygen effective cellular uptake rate	${\gamma }_{0}$	$0.85$ *mmHg/min*	optimization & ref. ^41^
Oxygen intravascular level	${\gamma }^{{in}}$	$60$ *mmHg*	^42,43^
Initial normoxia-hypoxia border distance	${\gamma }^{n-h}$	110 *μm*	^5,7^
Inactive drug effective diffusion coefficient	*D*_*ηi*_	*D*_*γ*_/50	compound size
Inactive drug vascular concentration	${\eta }_{i}^{0}$	50 *ag*/*μm*^3^	^17,21^
Inactive drug plasma clearance time	*t*_*ηi*_	$10$ *min*	^21^
Oxygen level for drug activation	${\gamma }_{{hypo}}$	$10$ *mmHg*	^24^
Drug activation function	${\varphi }^{{conv}}$	0.9/dt	high
Active drug effective diffusion coefficient	*D*_*ηa*_	*D*_*γ*_/25	compound size
Active drug decay rate	${\omega }_{a}$	0.1 log (2)/*min*	fitted
Active drug uptake rate	$\alpha$	$0.5$/*min*	fitted
Active drug lethal threshold	${\eta }_{a}^{{thr}}$	1 *ag*/*μm*^3^	^21^
Active drug vascular concentration	${\eta }_{a}^{0}$	0 *ag*/*μm*^3^	^21^
Sensitizer effective diffusion coefficient	*D*_*ξ*_	*D*_*γ*_	compound size
Enhancement rate	*ψ*_0_, *ψ*_1_, *ψ*_2_, *ψ*_3_	1, 5/3, 7.5/3, 12.5/3	^13^
for sensitizer levels	*ξ*_0_, *ξ*_1_, *ξ*_2_	9, 22, 88 *ag*/*μm*^3^	^13^
Sensitizer vascular concentration	*ξ*^*in*^	101 *ag*/*μm*^3^	^13^
Sensitizer plasma clearance time	*t*_*ξ*_	25 *min*	as vasodilator
Vasodilator-modulated flow decrease rate	*λ*	0.5	^17^
Vasodilator plasma clearance time	*t*_*λ*_	25 *min*	^17^

### Reporting summary

Further information on research design is available in the Nature Research Reporting Summary linked to this article.

### Supplementary information

Supplemental Material

Reporting summary

## Data Availability

The data from this study is available from the following depository: https://github.com/rejniaklab/HAP_schedules

## Code availability

The computational code used in this study is available from the following depository: https://github.com/rejniaklab/HAP_schedules
