# Supplementary material for: Exploring chronic and transient tumor hypoxia for predicting the efficacy of hypoxia-activated pro-drugs
Source: NPJ Syst Biol Appl. 2024 Jan 5;10:1. doi: 10.1038/s41540-023-00327-z (PMC10770176; doi:10.1038/s41540-023-00327-z)
Supplement: Supplementary file 1 — Supplemental Material [file 41540_2023_327_MOESM1_ESM.pdf]

Exploring chronic and transient tumor hypoxia for predicting the efficacy of hypoxia-activated pro-drugs

Shreya Mathur, Shannon Chen, Katarzyna A. Rejniak

Supplementary Methods. Computational implementation of the model

The model is defined on a two-dimensional (2D) domain ($\Omega = [0,200] \times [0,100] \mu m^2$) divided into 100×50 square grids of width $h = 2\mu m$. 365 individual cells were segmented from the histology image, as shown in Figure 6c in the main text. All cells are of different shapes and sizes, with areas between 4 and $844 \mu m^2$.

In order to calculate the interstitial fluid flow, we impose zero velocities on the domain boundaries: top $\mathbf{u}_{top} = [0,0]$ and bottom $\mathbf{u}_{bottom} = [0,0]$, and on cell boundaries: $\mathbf{u}_{cell} = [0,0]$, and the influx flow along the left domain boundary: $\mathbf{u}_{influx} = [1,0]$. If the forces $\mathbf{f}_k$ are known, the force-induced fluid velocity according to the regularized Stokeslets method, is given by Eq.(6) in the main text, and can be rewritten in the following way:

$$(1) \quad \mathbf{u}(\mathbf{x}) = \sum_{k=1}^{N} \left\{ -\frac{\mathbf{f}_k}{4\pi\mu} H_\varepsilon(r_k) + \frac{1}{4\pi\mu} [\mathbf{f}_k \cdot (\mathbf{x} - \mathbf{x}_k)](\mathbf{x} - \mathbf{x}_k) J_\varepsilon(r_k) \right\}, \quad r_k = \|\mathbf{x} - \mathbf{x}_k\|$$

$$\text{with} \quad (2) \quad H_\varepsilon(r) = \frac{1}{2} ln(r^2 + \varepsilon^2) - \frac{\varepsilon^2}{r^2 + \varepsilon^2} \quad \text{and} \quad J_\varepsilon(r) = \frac{1}{r^2 + \varepsilon^2}$$

where μ is the fluid viscosity. For the known velocities $\mathbf{u}(a_j, b_j)$, we need to calculate the unknown forces $\mathbf{f}_k$ at points $\mathbf{x}_k = (x_k, y_k)$, where $[\mathbf{f}_k] = [\mathbf{f}_{cell}, \mathbf{f}_{influx}, \mathbf{f}_{top}, \mathbf{f}_{bottom}]$, that yield that fluid flow. Thus, we solve the following matrix equation for $\mathbf{f}_k$ using an iterative GMRES algorithm (`gmres.m` in Matlab):

$$(3) \quad \mathbf{u}(a_j, b_j) = \sum_{k=1}^{N} M_{j,k}(\mathbf{x}_1, \cdots, \mathbf{x}_N) \mathbf{f}_k$$

$$\text{where} \quad (4) \quad M_{j,k} = \frac{1}{4\pi\mu} \begin{pmatrix} -H_\varepsilon(r) + (a_j - x_k)^2 J_\varepsilon(r) & (a_j - x_k)(b_j - y_k) J_\varepsilon(r) \\ (a_j - x_k)(b_j - y_k) J_\varepsilon(r) & -H_\varepsilon(r) + (b_j - y_k)^2 J_\varepsilon(r) \end{pmatrix}$$

The resulting fluid velocity flow is shown in Figure 6d in the main text. The zero-velocity values imposed on the cell boundaries guarantee that the compounds (oxygen, sensitizer, and active drug) carried via this advective transport will not cross the cell boundaries accidentally.

To ensure that the cell interior is not penetrated by the compounds unless they are directly absorbed via cell pseudo-receptors, the numerical implementation of the diffusive transport need to be modified to include only the grid points located in the interstitial space and omit these stencil points that are inside the cells. Therefore, the classical four-point stencil for the diffusion equation:

$$(5). \quad \gamma_{i,j}^{n+1} = \gamma_{i,j}^{n} + \frac{D_\gamma \, \Delta t}{h^2} \left(\gamma_{i-1,j}^{n} + \gamma_{i+1,j}^{n} + \gamma_{i,j-1}^{n} + \gamma_{i,j+1}^{n} - 4\, \gamma_{i,j}^{n} \right)$$

is thus replaced by the a non-penetrable stencil, where L is the number of stencil points that are located outside of the cells:

$$(6)\quad \gamma_{i,j}^{n+1} = \gamma_{i,j}^{n} + \frac{D_\gamma\,\Delta t}{h^2}\left(\sum_{k=1}^{L} \gamma_{i_k,j_k}^{n} - L\,\gamma_{i,j}^{n}\right)$$

To illustrate this concept, two cases of stencils for non-penetrable diffusion with some grid points located inside the cells are shown in the figure on the right. The appropriate equations are listed below:

$$(7)\quad \gamma_{k,l}^{n+1} = \gamma_{k,l}^{n} + \frac{D_\gamma\,\Delta t}{h^2}\left(\gamma_{k,l-1}^{n} + \gamma_{k,l+1}^{n} - 2\,\gamma_{k,l}^{n}\right)$$

$$(8)\quad \gamma_{m,p}^{n+1} = \gamma_{m,p}^{n} + \frac{D_\gamma\,\Delta t}{h^2}\left(\gamma_{m-1,p}^{n} + \gamma_{m+1,p}^{n} + \gamma_{m,p-1}^{n} - 3\,\gamma_{m,p}^{n}\right)$$

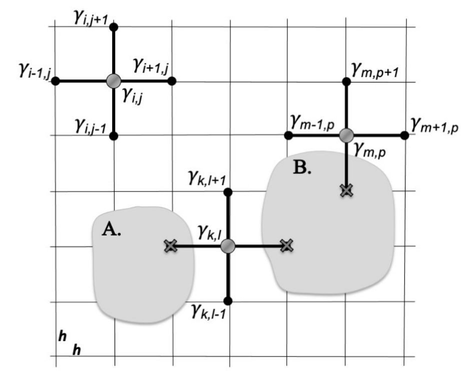

Non-penetrable diffusion stencil. Examples of a standard 4-point stencil (right-top corner) and two non-penetrable stencils (7, 8) for numerical implementation of the diffusion equation.

To ensure stability of the numerical scheme for diffusion, the following Courant-Friedrich-Lewy (CLF) condition much be satisfied: $D\Delta t/h^2 < 1/4$, where D is the diffusion coefficient, Δt is the time step, and h is the grid width. For our simulations, the time step was chosen to be $\Delta t = 0.15 \times 10^{-3}$ minutes to satisfy the CLF condition for all diffusive compounds.

Cellular uptake of the diffusive compounds is calculated from four grid points surrounding each of the points belonging to the given cell. To assure that the compound concentration does not attain a negative value, the cellular uptake rate α must satisfy the following condition: $1 - \alpha\Delta t > 0$. Since the cells are of different sizes, the lethal threshold for active drug is determined by normalizing the absorbed drug level by the cell 's area.

This mathematical model has been implemented in the MATLAB system and the associated code is available at the GitHub depository system (https://github.com/rejniaklab/HAP_schedules).

Supplementary Figure 1.

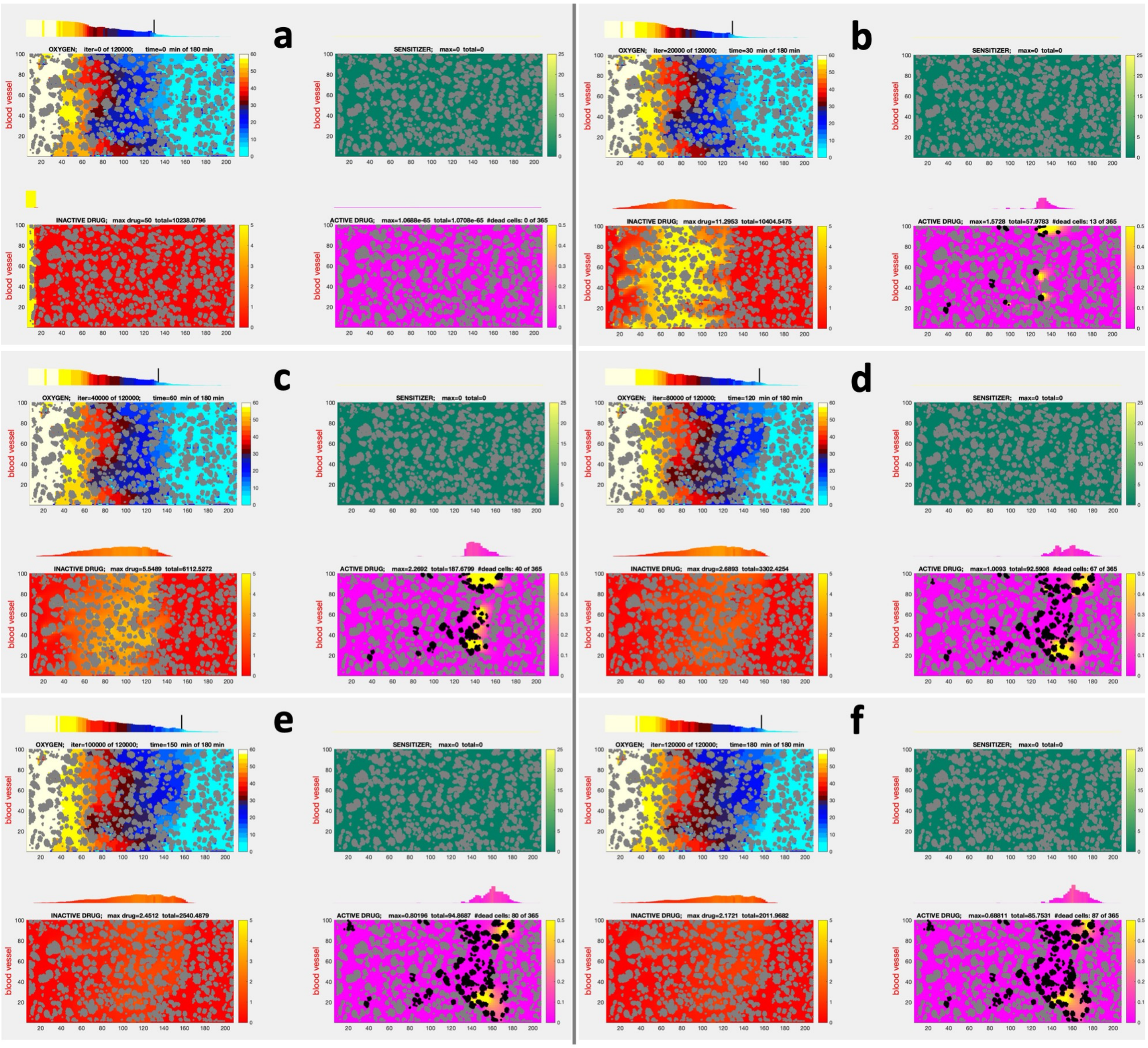

Supplementary Figure 1. Snapshots from a simulation of HAP monotherapy. A time series of snapshots from a simulation of a single bolus injection of HAP at time 0. The HAP was administered at time 0; the four panels in each snapshot show tissue oxygenation (top-left), sensitizer distribution (top-right), distribution of the inactive drug (bottom-left), distribution of the active drug and dead cells in black (bottom-right) at times: **a.** 0 hours; **b.** 0.5 hour; **c.** 1 hour; **d.** 2 hours; **e.** 2.5 hours; **f.** 3 hours. Final number of dead cells: 87 out of 365 (23.8%).

Supplementary Figure 2.

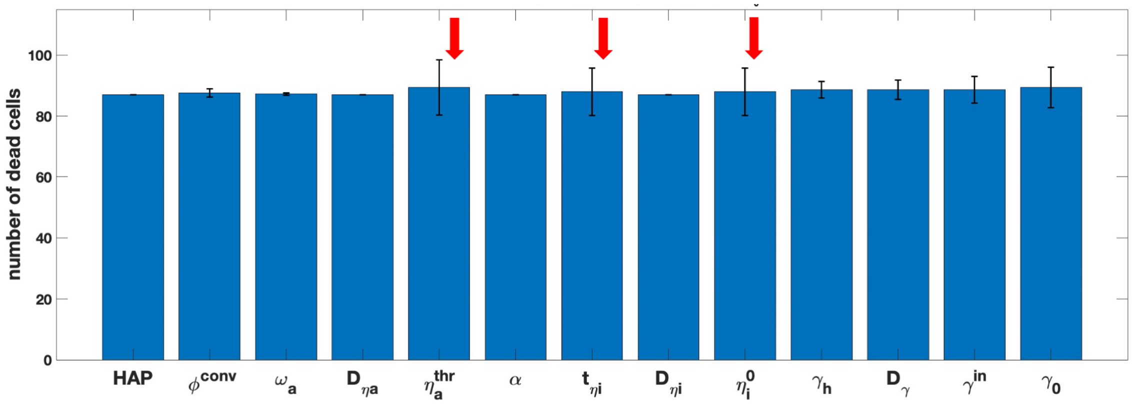

Supplementary Figure 2. Local sensitivity analysis of model parameters for the HAP monotherapy. For the HAP monotherapy (HAP administered at time 0, results in Supplementary Figure 1), twelve parameters were considered, and each parameter was varied within a +/- 20% range of the baseline value (in 10% increments). The average +/- standard deviation values are presented. The histogram shows the final number of death cells for: (i) the baseline HAP data (87 dead cells); (ii) drug activation rate, φ^{conv} (87.6 dead cells, std=1.34); (iii) active drug decay, ω_a (87.2 dead cells, std=0.44); (iv) diffusion coefficient of an active drug, $D_{\eta a}$ (87 dead cells, std=0); (v) lethal threshold of an active drug, η_a^{thr} (89.4dead cells, std=9.13); (vi) cellular uptake of an active drug, α (87 dead cells, std=0); (vii) inactive drug plasma clearance time $t_{\eta i}$ (88 dead cells, std=7.78); (viii) diffusion coefficient of an inactive drug, $D_{\eta i}$ (87 dead cells, std=0); (ix) inactive drug vascular concentration, η_i^0 (88 dead cells, std=7.78); (x) oxygen level for HAP activation, γ_{hypo} (88.6 dead cells, std=2.7); (xi) diffusion coefficient of oxygen, D_γ (88.6 dead cells, std=3.2); (xii) oxygen intravascular concentration, γ^{in} (88.6 dead cells, std=4.4); (xiii) oxygen uptake rate, γ_0 (89.4 dead cells, std=6.65). The vertical lines indicate standard deviation values. The parameters with a standard deviation above 7.5 are indicated by red arrows and are defined as sensitive.

Supplementary Figure 3.

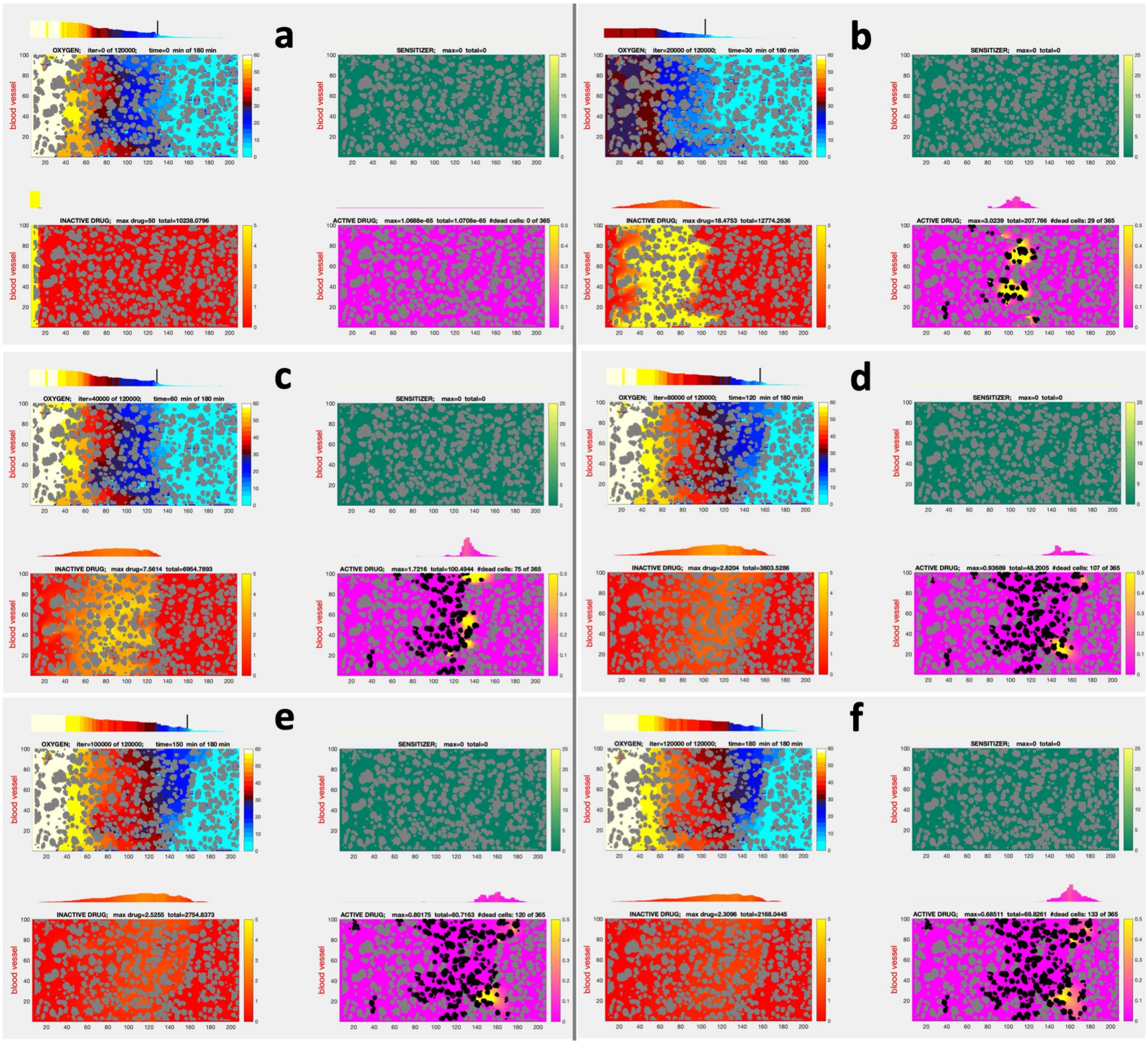

Supplementary Figure 3. Snapshots from a simulation of HAP+Vaso combination therapy. A time series of snapshots from a simulation of a combination of HAP and a vasodilator Vaso, with HAP administered at time 0, and Vaso 10 minutes later. The four panels in each snapshot show tissue oxygenation (top-left), sensitizer distribution (top-right), distribution of the inactive drug (bottom-left), distribution of the active drug and dead cells in black (bottom-right) at times: **a.** 0 hours; **b.** 0.5 hour; **c.** 1 hour; **d.** 2 hours; **e.** 2.5 hours; **f.** 3 hours. Final number of dead cells: 133 out of 365 (36.4%).

Supplementary Figure 4.

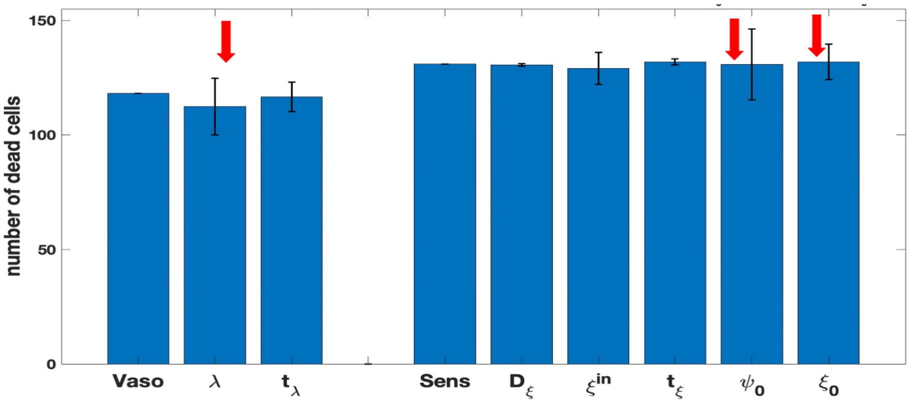

Supplementary Figure 4. Local sensitivity analysis of model parameters for the HAP+Vaso and HAP+Sens combination therapies. For the combination therapy of HAP+Vaso (HAP administered at time 0 and Vaso at 10 minutes, results in Supplementary Figure 3) and HAP+Sens (Sens administered at time 0 and HAP at 20 minutes, results in Supplementary Figure 5), nine parameters were considered, and each was varied within a +/- 20% range of the baseline value (in 10% increments). The average +/- standard deviation values are presented. The histogram shows the final number of death cells HAP+Vaso therapy (left) and HAP+Sens therapy (right) with schedules from Supplementary Figure 3 and Supplementary Figure 5, respectively. Parameters considered: (i) the baseline HAP+Vaso data (118 dead cells); (ii) vasodilator-modulated decrease in influx rate, λ (112.4 dead cells, std=12.4); (iii) vasodilator plasma clearance time, t_λ (116.6 dead cells, std=6.5); (iv) the baseline HAP+Sens data (131 dead cells); (v) sensitizer diffusion coefficient,D_ξ (130.6 dead cells, std=0.55); (vi) sensitizer vascular concentration, ξ^{in} (129 dead cells, std=6.96); (vii) sensitizer plasma clearance time, t_ξ (131.8 dead cells, std=1.3); (viii) the sensitizer-modulated enhancement rate of oxygen uptake, ψ_0 (130.8 dead cells, std=15.5); (ix) the sensitizer level for enhancement of the oxygen uptake rate, ξ_0 (131.8 dead cells, std=7.7). The vertical lines indicate standard deviation values. The parameters with a standard deviation above 7.5 are indicated by red arrows and defined as sensitive.

Supplementary Figure 5.

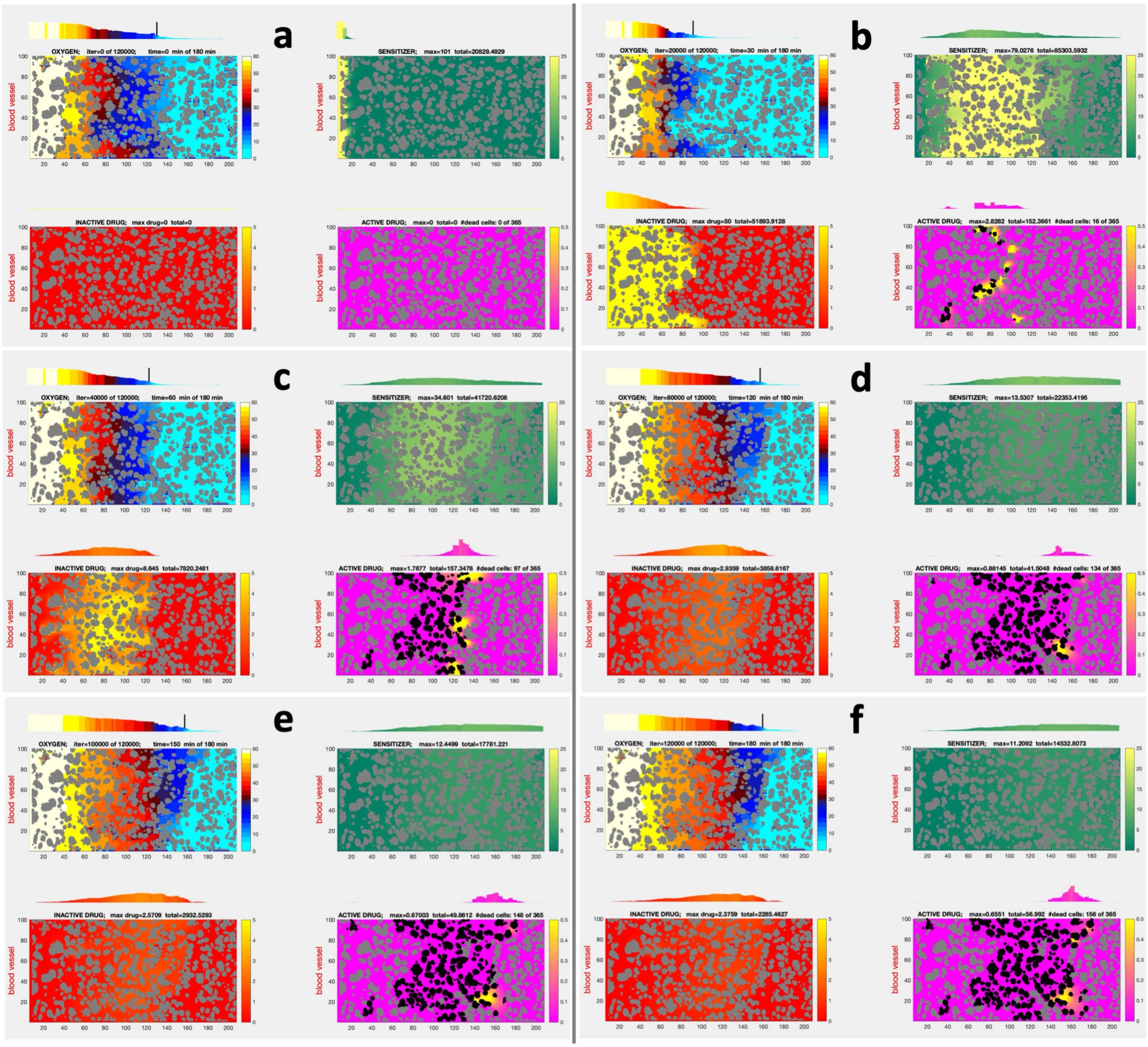

Supplementary Figure 5. Snapshots from a simulation of HAP+Sens combination therapy. A time series of snapshots from a simulation of a combination of HAP and a sensitizer Sens, with Sens administered at time 0, and HAP 20 minutes later. The four panels in each snapshot show tissue oxygenation (top-left), oxygen sensitizer distribution (top-right), distribution of the inactive drug (bottom-left), distribution of the active drug and dead cells in black (bottom-right) at times: **a.** 0 hours; **b.** 0.5 hour; **c.** 1 hour; **d.** 2 hours; **e.** 2.5 hours; **f.** 3 hours. Final number of dead cells: 156 out of 365 (42.7%).

Supplementary Figure 6.

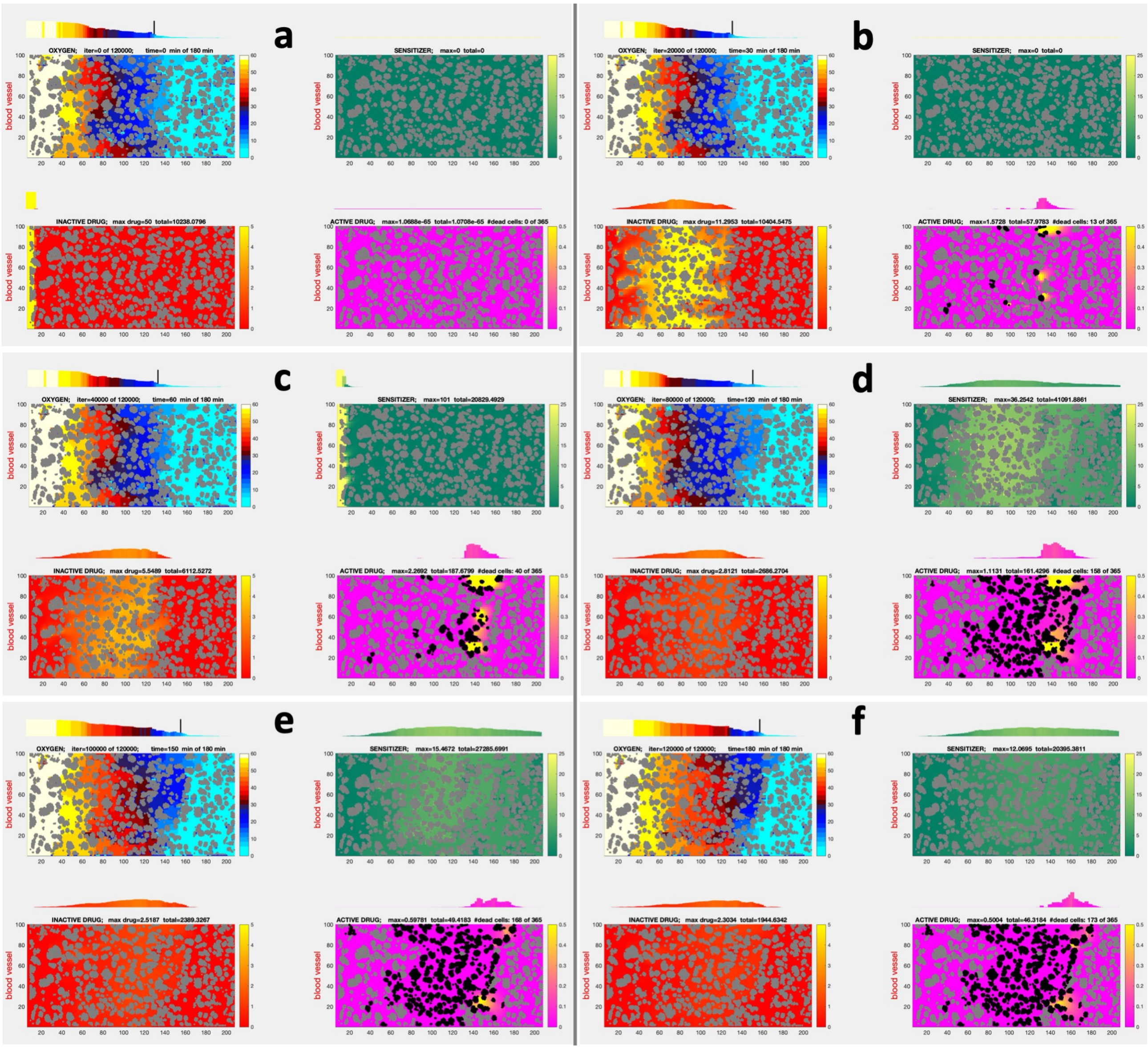

Supplementary Figure 6. Snapshots from a simulation of HAP+Vaso+Sens combination therapy with an optimal schedule. A time series of snapshots from a simulation of a combination of HAP with a vasodilator Vaso, and a sensitizer Sens. HAP was administered at time 0, Sens 60 minutes after HAP, and Vaso 80 minutes after HAP. The four panels in each snapshot show tissue oxygenation (top-left), oxygen demand sensitizer distribution (top-right), distribution of the inactive drug (bottom-left), distribution of the active drug and dead cells in black (bottom-right) at times: **a.** 0 hours; **b.** 0.5 hour; **c.** 1 hour; **d.** 2 hours; **e.** 2.5 hours; **f.** 3 hours. Final number of dead cells: 173 out of 365 (47.4%).

Supplementary Figure 7.

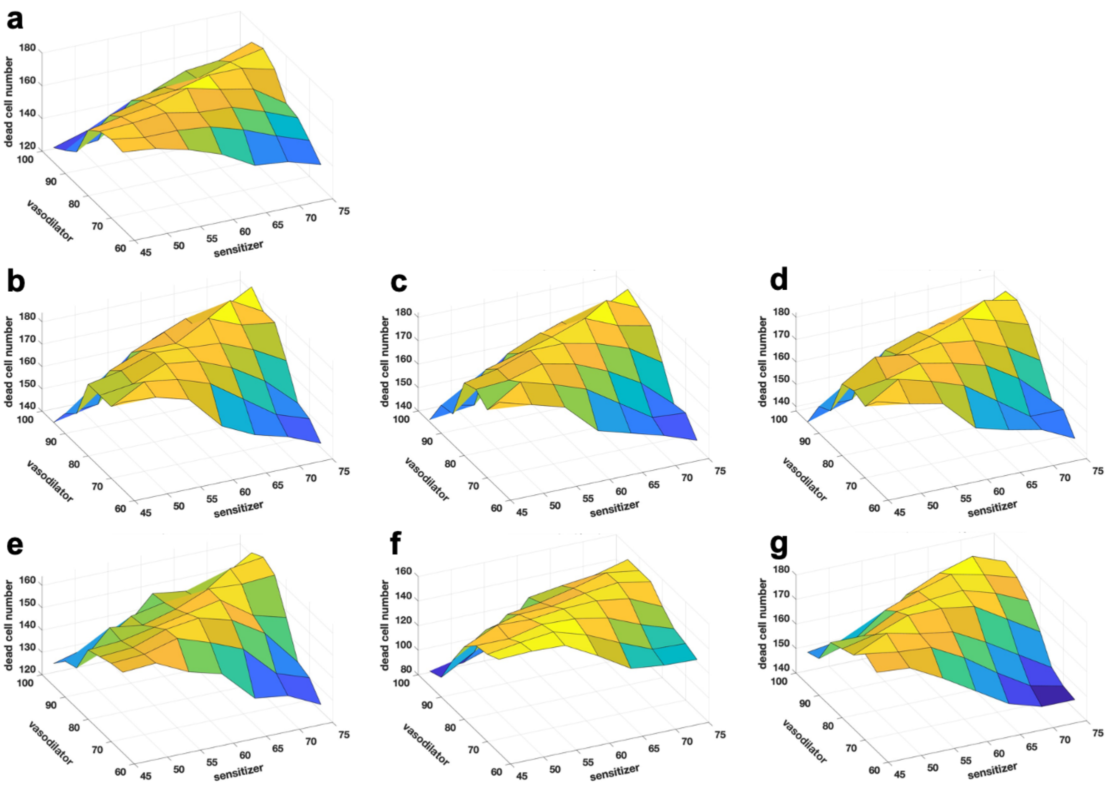

Supplementary Figure 7. Analysis of optimal schedule robustness to changes in model parameters. For the robustness analysis of combination therapy HAP+Vaso+Sens (HAP administered at time 0, Sens 60 minute later, and Vaso 80 minutes after HAP, results in Supplementary Figure 6), six sensitive parameters were considered. The surface plots show the final number of death cells after completion of the three-compound therapy where the time of each compound injection was varied within +/- 15 minutes (with 5-minutes increments) from the most effective administration schedule from Supplementary Figure 6. **A.** The original data (subset of Figure 5a in the main text) with maximal number of dead cells: 173 for a schedule with Sens=60 and Vaso 80 minutes after HAP. **B-G.** Six sensitive parameters fixed at 20% deviation from the baseline value, as identified in Supplementary Figures 2 and 4. **B.** Lethal threshold of an active drug, η_a^{thr} (184 dead cells for Sens=65, Vaso=85; 3% difference from schedule Sens=60, Vaso=80, that results in 179 deaths); **C.** Inactive drug plasma clearance time $t_{\eta i}$ (182 dead cells for Sens=65, Vaso=85; 2% difference from Sens=60, Vaso=80, that results in 179 deaths); **D.** Inactive drug vascular concentration, η_i^0 (184 dead cells for Sens=65, Vaso=85; 3% difference from Sens=60, Vaso=80, that results in 179 deaths); **E.** Vasodilator-modulated decrease in influx rate, λ (164 dead cells for Sens=70, Vaso=95; 4% difference from Sens=60, Vaso=80, that results in 158 deaths); **F.** The sensitizer-modulated enhancement rate of oxygen uptake, ψ_0 (158 dead cells for Sens=50, Vaso=75; 3% difference from Sens=60, Vaso=80, that results in 153 dead cells); **G.** the sensitizer level for enhancement of the oxygen uptake rate, ξ_0 (178 dead cells for Sens=70, Vaso=85; 1% difference from Sens=60, Vaso=80, that results in 176 dead cells); Based on the results from 49 simulations run for each parameter, the most efficient schedules in each set of simulations were only 1-4% different from the corresponding results for the HAP=0, Sens=60, and Vaso=80 schedule. These differences are insignificant, and the HAP=0, Sens=60, and Vaso=80 schedule is robust in respect to all sensitive parameters.

Supplementary Figure 8.

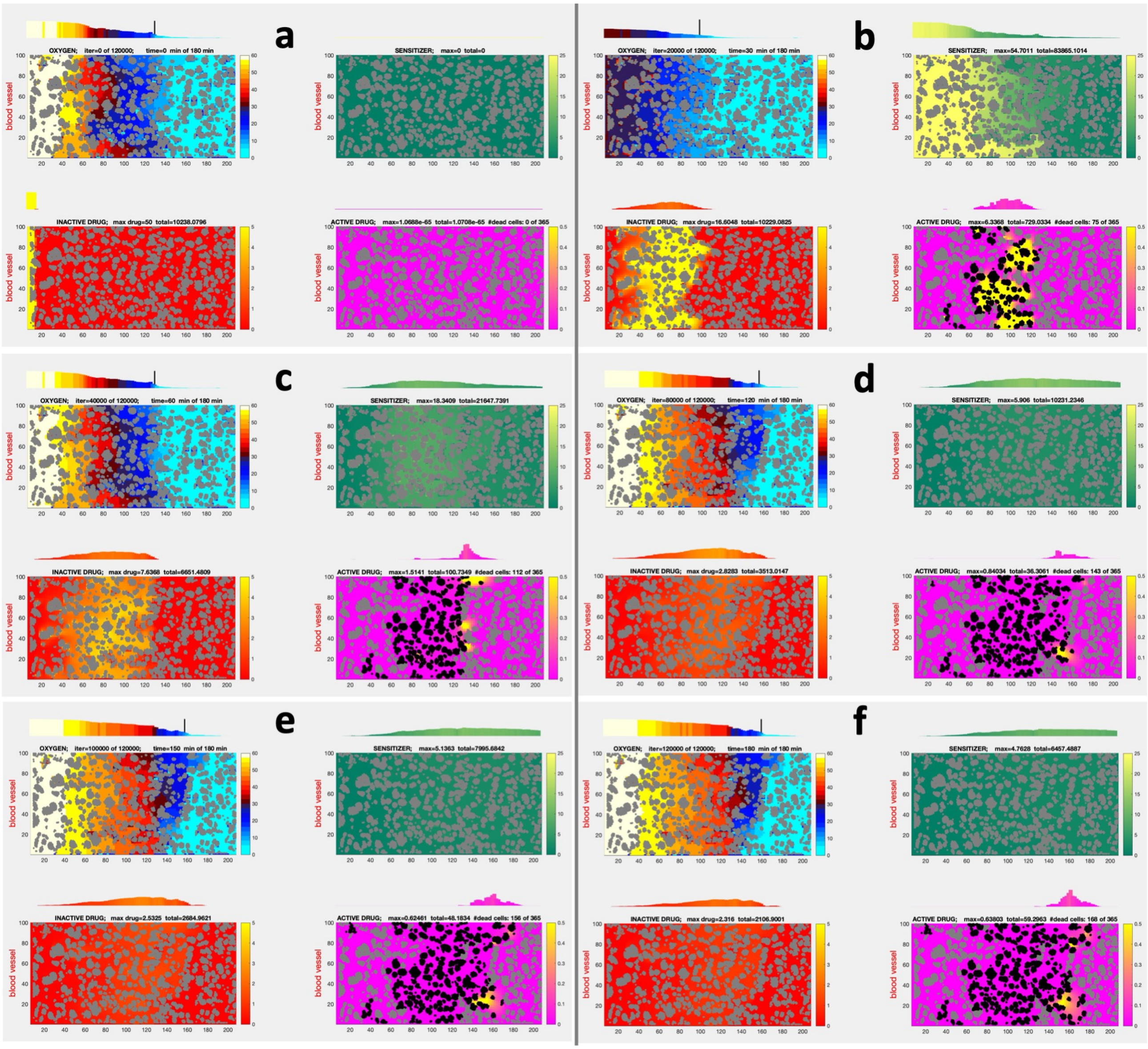

Supplementary Figure 8. Snapshots from a simulation of HAP+Vaso+Sens combination therapy with a locally optimal schedule A. A time series of snapshots from a simulation of a combination of HAP with a vasodilator Vaso, and a sensitizer Sens, with HAP administered at time 0, Sens 10 minutes later, and Vaso 15 minutes after HAP. The four panels in each snapshot show tissue oxygenation (top-left), oxygen demand sensitizer distribution (top-right), distribution of the inactive drug (bottom-left), distribution of the active drug and dead cells in black (bottom-right) at times: **a.** 0 hours; **b.** 0.5 hour; **c.** 1 hour; **d.** 2 hours; **e.** 2.5 hours; **f.** 3 hours. Final number of dead cells: 168 out of 365 (46.0%).

Supplementary Figure 9.

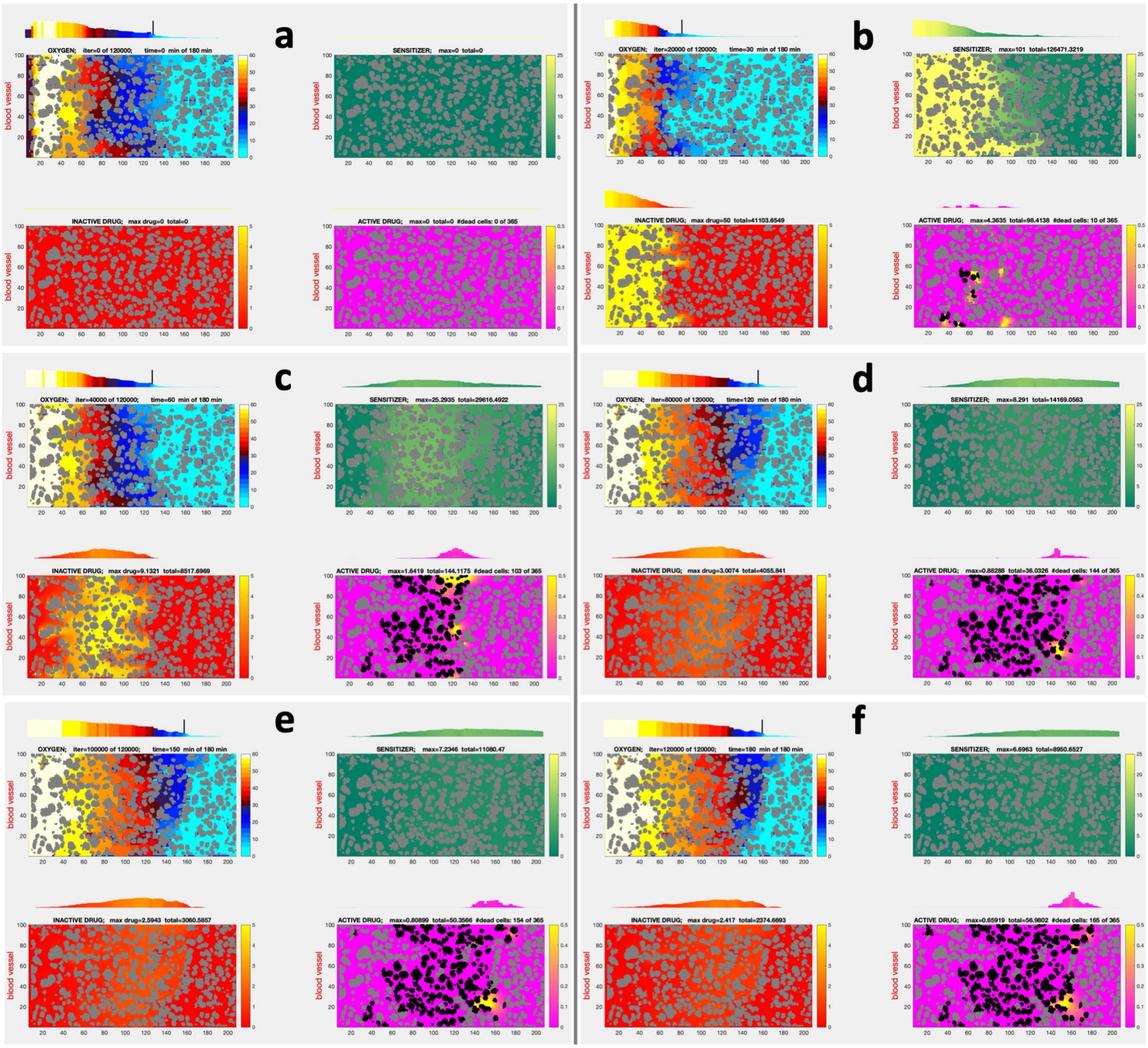

Supplementary Figure 9. Snapshots from a simulation of HAP+Vaso+Sens combination therapy with a locally optimal schedule B. A time series of snapshots from a simulation of a combination of HAP with a vasodilator Vaso, and a sensitizer Sens, with Vaso administered at time 0, Sens 10 minutes later, and HAP 25 minutes after Vaso. The four panels in each snapshot show tissue oxygenation (top-left), oxygen demand sensitizer distribution (top-right), distribution of the inactive drug (bottom-left), distribution of the active drug and dead cells in black (bottom-right) at times: **a.** 0 hours; **b.** 0.5 hour; **c.** 1 hour; **d.** 2 hours; **e.** 2.5 hours; **f.** 3 hours. Final number of dead cells: 165 out of 365 (45.2%).
